# Dual synthesis pathways of scaRNA28 via intronic processing of transformation/transcription domain-associated protein transcripts and a novel independent transcription unit

**DOI:** 10.1080/15476286.2025.2513133

**Published:** 2025-05-30

**Authors:** Keiichi Izumikawa, Tatsuya Shida, Hideaki Ishikawa, Sotaro Miyao, Takayuki Ohga, Masato Taoka, Yuko Nobe, Hiroshi Nakayama, Masami Nagahama

**Affiliations:** a Laboratory of Molecular and Cellular Biochemistry, Meiji Pharmaceutical University, Kiyose-shi, Japan; b Department of Applied Biological Science & Global Innovation Research Organizations, Tokyo University of Agriculture and Technology, Tokyo, Japan; c Department of Chemistry, Graduate School of Science, Tokyo Metropolitan University, Tokyo, Japan; d Biomolecular Characterization Unit, RIKEN Center for Sustainable Resource Science, Saitama, Japan

**Keywords:** scaRNA, snoRNA, MYC, non-coding RNA, independent transcription unit

## Abstract

Small Cajal body-specific RNAs (scaRNAs) are noncoding RNAs involved in the maturation of U-rich small nuclear RNAs. Except for a few that have their own transcription units, most scaRNA genes are embedded in introns and are predicted to be transcribed with host genes. Herein, we report that scaRNA28 is the first scaRNA with a dual synthesis pathway, and that this RNA is transcribed in an independent transcription unit (ITU) by RNA polymerase II while located in intron 2 of the transformation/transcription domain-associated protein (TRRAP) gene. We evaluated the scaRNA28 synthesis pathway using minigenes containing exon 2, intron 2, and exon 3 of *TRRAP*. A minigene with a mutation preventing 5′ splicing recognition of the exon 2/intron 2 junction generated scaRNA28, suggesting a pathway processing unspliced transcripts into scaRNA28. Even promoterless minigenes and DNA fragments with regions from exons 2 to 3 of *TRRAP* showed RNA polymerase II-dependent synthesis of scaRNA28, indicating a novel synthesis pathway involving an ITU. Linker-scanning mutational analysis revealed that the promoter region required for scaRNA28 expression in the ITU is located within 60 bases including exon 2/intron 2 junction of *TRRAP*, and especially the first two bases of intron 2 region, a putative part of the MYC-binding (E-box) motif, are essential for scaRNA28 expression in the ITU. MYC promotes scaRNA28 expression by binding to the promoter region in the ITU. Our findings demonstrated a novel transcriptional pathway for the synthesis of scaRNA28, the first scaRNA with a dual synthesis pathway.

## Introduction

In eukaryotes, pre-mRNA splicing is a crucial biological process that substantially contributes to generating several protein repertoires relative to the number of genes in an organism, imparting diverse characteristics to each tissue. The spliceosome, the functional complex responsible for pre-mRNA splicing, consists of U1, U2, U4, U5, and U6 small nuclear RNAs (snRNAs) in the major spliceosome, along with U11, U12, U4atac, U5, and U6atac in minor splicing, in addition to numerous interacting proteins [1,2]. During the biogenesis processes of U snRNAs, each snRNA is transcribed as an immature precursor snRNA and sequentially processed through the trimming of the 3′ end and internal chemical modifications [3–6]. Most chemical modifications in the internal sequence of U snRNAs occur in Cajal bodies, nuclear membraneless organelles in which fibrillarin is responsible for 2′-*O*-methylation and dyskerin for pseudouridylation of U snRNAs [7]. Small Cajal body-specific RNAs (scaRNAs) are noncoding RNAs involved in the maturation of U snRNAs. During the chemical modification processes of U snRNAs, scaRNAs form protein-RNA complexes with the enzymes for 2′-*O*-methylation or pseudouridylation and serve as guide RNAs to precisely select specific sites which are chemically modified in the U snRNA [8]. scaRNAs and related complexes containing modification enzymes are localized to the Cajal body, and the proper localization of scaRNA to the Cajal body is essential for the accurate chemical modification of U-snRNA [9–11]. The underlying mechanism is thought to involve TCAB1, TDP-43, or Nopp140 transporting scaRNAs to the Cajal body by binding to the internal CAB box or UG repeat sequences of the scaRNAs [8,10–12].

To date, at least two scaRNA synthesis pathways have been identified. One is an intronic-embedded scaRNA, and the other is a scaRNA with independent transcription units (ITUs), such as scaRNA2, scaRNA17, or scaRNA19 [13,14]. scaRNA2, scaRNA17, and scaRNA19 (known as telomerase RNA; TR or TERC) have ITUs and are transcribed by RNA polymerase II [15–17]; these transcripts are modified with methylated guanosine cap structure at the 5′-terminus of RNA moieties in a transcriptionally coupled manner [17,18]. Other scaRNAs are predicted to be intronic-embedded scaRNAs, and the transcription of host genes and subsequent splicing and processing of introns generate each scaRNA [14]. Addition of oligo(A) tail by noncanonical poly(A) polymerase PAPD5 and deadenylation by poly(A) specific ribonuclease PARN are involved at the 3' end processing of some scaRNAs. However, the detailed molecular mechanisms underlying the synthesis of many other scaRNAs remain elusive [19]. Changes in scaRNA and small nucleolar RNA (snoRNA) expression have been recently observed in many cancer cells and tumours using transcriptome analysis; however, the molecular mechanisms underlying these changes are not fully understood [20–22].

scaRNA28 is a scaRNA with a C/D box motif and is responsible for the 2′-*O*-methylation of U47 in U2 snRNA [10,11]. It contains a UG repeat in its internal sequence, which is necessary for its localization to Cajal bodies via TDP-43 or Nopp140 binding [11,12]. As *SCARNA28* is in intron 2 of the transformation/transcription domain-associated protein (*TRRAP*), it is predicted to be a typical intronic-embedded scaRNA. In this study, we investigated the synthesis of scaRNA28 and identified an unexpected synthesis pathway of scaRNA28 using several minigenes or DNA fragments consisting of exon 2, intron 2, and exon 3 of *TRRAP* to evaluate scaRNA28 expression. In addition to the synthetic pathway that processes the intronic sequences of host gene transcripts, we found that scaRNA28 has an ITU that is dependent on transcription by RNA polymerase II, which differs from host gene expression. To the best of our knowledge, this is the first report of a scaRNA with dual synthesis pathways.

## Materials and methods

### Antibodies and reagents

Antibodies used in this study are listed in Supplementary Table S1. A stabilized streptavidin-horseradish peroxidase (HRP) conjugate (Thermo Fisher Scientific, USA, 89880D) was used to detect biotinylated oligonucleotides or proteins. All the reagents were purchased from Wako Pure Chemical Industries (Osaka, Japan), Kanto Chemical Co. (Tokyo, Japan), or Nacalai Tesque (Kyoto, Japan).

### Cell culture

MCF7 and 293T cells (HEK293 cells transformed with the large T antigen) were cultured in Dulbecco’s modified Eagle’s medium (DMEM; Sigma-Aldrich). Flp-In T-REx 293 (293FTR) cells (Thermo Fisher Scientific) were cultured in Dulbecco’s modified Eagle medium with high glucose (Wako Pure Chemical Industries, Osaka, Japan). All cell lines were cultured at 37 °C under 5 % CO_2_ in air. All media were supplemented with 10 % heat-inactivated foetal bovine serum (Biowest LLC), streptomycin (0.1 mg/mL), and penicillin G (100 U/mL). α-amanitin or ML-60218 (Sigma-Aldrich 557403) were used for 12 h at concentrations of 5 μg/mL and 40 μM, respectively.

### Construction of expression plasmids

The construction of a plasmid expressing scaRNA28 (pcDNA5/FRT/TO DAP-tagged TRRAP Exon2-Intron2-Exon3 (DAP-Ex2-Int2-Ex3)) has been previously described [11]. The sequence of DAP-tag was indicated in Supplementary Figure S1. To construct a plasmid expressing DAP-Ex2-Ex3, DNA fragments encoding TRRAP Exon2-Exon3 (NM_001375524:168–317) were amplified using KOD Plus Neo (TOYOBO, Japan) with a primer set (KIT-1/KIT-2) using cDNA derived from 293T cells as template DNA. The amplified DNA was digested with BamHI and XhoI and inserted into the BamHI and XhoI sites of pcDNA5/FRT/TO DAP-TDP-43 as previously described [11]. pcDNA5/FRT/TO DAP-Ex2-Int2-Ex3 dSp1 or pcDNA5/FRT/TO DAP-Ex2-Int2-Ex3-y18Sn was amplified using KOD Plus Neo with primer sets (KI-115/KI-116 or KI-92/KI-93) using pcDNA5/FRT/TO DAP-Ex2-Int2-Ex3 as DNA template. pcDNA5/FRT/TO DAP-Ex2-Int2-Ex3 dSp2 or pcDNA5/FRT/TO DAP-Ex2-Int2-Ex3-y18Sn dSp3 were amplified using KOD Plus Neo with primer sets (KI-374/KI-375) using pcDNA5/FRT/TO DAP-Ex2-Int2-Ex3 dSp1 or DAP-Ex2-Int2-Ex3-y18Sn as DNA template. The template DNA was degraded by digestion with DpnI after PCR. To construct pcDNA5/FRT/TO DAP-Ex2-Int2-Ex3 p(CU)_11_, DNA fragments were amplified using KOD Plus Neo with primer sets (KIT-5 and KIT-6) using pcDNA5/FRT/TO DAP-Ex2-Int2-Ex3 as a template DNA, which was degraded with DpnI after PCR. The amplified DNA was treated with T4 Polynucleotide kinase and self-ligated using T4 DNA ligase (Takara Bio, Japan). To construct promoterless minigenes, the CMV promoter sequence was removed from pcDNA5/FRT/TO by BglII digestion and self-ligated with T4 DNA ligase to construct pcDNA5/FRT/TO-dCMV. To construct pcDNA5/FRT/TO-dCMV DAP-Ex2-Int2-Ex3 and pcDNA5/FRT/TO-dCMV DAP-Ex2-Int2-Ex3-y18Sn, DNA fragments of DAP-Ex2-Int2-Ex3 and DAP-Ex2-Int2-Ex3-y18Sn were amplified using KOD FX Neo (TOYOBO, Japan) with primer sets (KIT-1/KIT-2) using pcDNA5/FRT/TO DAP-Ex2-Int2-Ex3 and pcDNA5/FRT/TO DAP-Ex2-Int2-Ex3-y18Sn as template DNA, respectively. The amplified DNA was digested with BamHI and XhoI and inserted into the BamHI and XhoI sites of pcDNA5/FRT/TO-dCMV. To construct the deletion mutant series of pcDNA5/FRT/TO-dCMV DAP-Ex2-Int2-Ex3-y18Sn (Δ101–200, Δ201–300, Δ301–400, Δ401–500, Δ101–140, Δ121–160, Δ141–180, Δ161–200, and Δ121–500), DNA fragments were amplified using KOD FX Neo with primer sets (KI-195/KI-196 for Δ101–200, KI-197/KI-198 for Δ201–300, KI-199/KI-200 for Δ301–400, KI-201/KI-202 for Δ401–500, KI-195/KI-209 for Δ101–140, KI-210/KI-211 for Δ121–160, KI-212/KI-213 for Δ141–180, KI-214/KI-196 for Δ161–200, and KI-202/KI-210 for Δ121–500) using pcDNA5/FRT/TO-dCMV DAP-Ex2-Int2-Ex3-y18Sn as DNA template. The template DNA was degraded by digestion with DpnI after PCR, and the amplified DNA was treated with T4 Polynucleotide kinase and self-ligated with T4 DNA ligase. pcDNA5/FRT/TO-dCMV DAP-Ex2-Int2-Ex3-y18Sn pEb-1 and pcDNA5/FRT/TO-dCMV DAP-Ex2-Int2-Ex3-y18Sn pEb-2 were amplified using KOD FX Neo with primer sets (KI-215/KI-216 and KI-115/KI-116) and pcDNA5/FRT/TO-dCMV DAP-Ex2-Int2-Ex3-y18Sn as DNA template. The template DNA was degraded by digestion with DpnI after PCR. To construct pcDNA3.1(+) HA-MYC, DNA fragments were amplified using KOD Plus Neo with primer sets (KIT-7/KIT-8) and cDNA from 293T cells as the template. The PCR fragments were digested with BamHI and XhoI and inserted into the BamHI and XhoI sites of HA-tag-fused pcDNA3.1(+), as previously described [23]. DNA fragments encoding MYC were excised from pcDNA3.1 (+) HA-MYC using BamHI and XhoI digestion and inserted into the BamHI and XhoI sites of FLAG-tagged pcDNA5/FRT/TO, which were generated using BamHI and XhoI digestion of pcDNA5/FRT/TO FLAG-Chtop [23]. The resulting plasmid was named as pcDNA5/FRT/TO FLAG-MYC. Construction of pRcU6PT, which encodes only RAT-tagged RNA, has been previously described [24]. All plasmid sequences were confirmed using DNA sequencing. Primers used in this study are listed in Supplementary Table S2. All plasmids used in this study are listed in Supplementary Table S3.

### Transfection

Flp-In T-REx 293 and 293T cells were transfected with the indicated minigenes or DNA fragments using Lipofectamine 2000 (Thermo Fisher Scientific) according to the manufacturer’s instructions. Briefly, cells in a twelve-well plate were transfected with a DNA solution containing 160 ng of the minigene or 100 ng of the DNA fragment and 160 ng of pRcU6PT as a transfection control. Cells were harvested 24 h after transfection and were subjected to subsequent experiments. All siRNA sequences are listed in Supplementary Table S2.

### Northern blotting

Total RNA was isolated using the TRIzol Reagent (Thermo Fisher Scientific, USA). The isolated RNA was treated with DNase I RT-Grade (Nippon Gene, Japan) at 37 °C for 30 min and extracted with Phenol Chloroform extraction, and isopropanol precipitation. Total RNA (4 or 5 μg) was subjected to denaturing (8 M) urea 7% PAGE in Tris-borate-EDTA running buffer, stained with SYBR Gold (Invitrogen, USA) for 10 min, and then visualized using the LAS4000 Luminescent Image Analyzer System (Fujifilm, Japan). The separated RNAs were transferred to a Hybond N+ membrane (GE Healthcare, USA) at 10 V for 60 min using Transblot Turbo system (BIO-RAD, Japan). The membrane was dried and subsequently cross-linked using an ultraviolet cross linker (Funakoshi, Tokyo, Japan) at 120 mJ/cm^2^. Hybridization with biotin-labelled DNA probes was performed using PerfectHyb Plus (Sigma-Aldrich) or ExpressHyb Hybridization Solution (Takara Bio, Japan) according to the manufacturer’s instructions. Chemiluminescent signals of the RNA bands were detected using the LAS-4000 system or FUSION (M&S Instruments Inc., Osaka, Japan). Signal intensities were quantified using the Image Quant TL software (GE Healthcare) or ImageJ-2 (NIH). The biotin-labelled DNA oligonucleotide probes used in this study are listed in Supplementary Table S2.

### RT-PCR

Total RNA was isolated using the TRIzol Reagent (Thermo Fisher Scientific, USA). The isolated RNA was treated with DNase I RT-Grade (Nippon Gene) at 37 °C for 30 min and extracted with TRIzol Reagent. Reverse transcription was performed with oligo dT primers using the PrimeScript RT-PCR Kit (Takara Bio) or ReverTra Ace (TOYOBO). PCR was performed using TaKaRa Ex Taq DNA polymerase (Takara Bio) with the primer set (KIT-10/KIT-11) on a Thermal Cycler Dice (Takara Bio) or a GeneAmp PCR System 9700 thermocycler (Applied Biosystems, USA). DNA sequences of the primers used in this study are listed in Supplementary Table S2.

### Western blotting

Immunoblotting was performed as previously described [11]. Biotinylated proteins were detected using a stabilized streptavidin HRP Conjugate (Thermo Fisher Scientific). The chemiluminescent signals of the protein bands were detected using a LAS-4000 system (GE Healthcare) or FUSION. The antibodies used in this study are listed in Supplementary Table S1.

### Preparation of DNA fragments for scaRNA28 or y18Sn-scaRNA28 expression

Ten nanograms of pcDNA5/FRT/TO DAP-Ex2-Int2-Ex3 or the indicated promoterless minigenes (e.g. pcDNA5/FRT/TO-dCMV DAP-Ex2-Int2-Ex3) were digested with BamHI for 1 h at 37 °C and then purified by PCR purification kit (Qiagen, USA). Of these, 1 ng equivalent of linearized minigenes was used as template DNA, and DNA fragments were amplified using KOD FX Neo with indicated primer sets described in Supplementary Table S4. The amplified DNA fragments were purified by gel extraction on 1.5 % agarose gel using a MinElute PCR Purification Kit (Qiagen). Primers used in this study are listed in Supplementary Table S2. The minigenes selected as template DNA and primer sets used for PCR to generate DNA fragments are listed in Supplementary Table S4.

### Immunoprecipitation of 7-methylguanosine (m^7^G)-capped RNA

Anti-m^7^G antibody-bound beads were prepared by incubating 10 μg of antibody and 50 μl of protein G Dynabeads (Invitrogen) with 50 mM Tris-HCl pH7.4, 150 mM NaCl, and 0.01% Triton X-100 for 30 min at 25°C, and then washing three times with 50 mM Tris-HCl pH7.4, 150 mM NaCl and 0.01% Triton X-100. Total RNA was extracted from cells expressing y18Sn-scaRNA28 derived from Ex2-Int2-Ex3-y18Sn minigene as described above. m^7^G-capped RNA were immunoprecipitated by rotating 200 μg of total RNA and anti-m^7^G antibody beads in 500 μl of the binding solution [50 mM Tris-HCl pH7.4, 150 mM NaCl, 0.01% Triton X-100, and 40 U of recombinant RNase inhibitor ver.2.0 (Takara Bio)] for 4 h at 4 °C. Antibody-bound beads were washed five times with 500 μl of the binding solution, and m^7^G-capped RNA was extracted from antibody-bound beads using TRIzol reagent, as described above. The obtained m^7^G-capped RNA was subjected to reverse transcription with random hexamers using ReverTra Ace according to the manufacturer’s instructions. y18Sn-scaRNA precursors with m^7^G cap were detected by PCR using the primer sets amplifying the region from intron 2 of TRRAP to y18Sn tag. Mouse IgG2a was used as the control. Primer sequences used in this study are listed in Supplementary Table S2. The antibodies used in this study are listed in Supplementary Table S1.

## Results

### Construction of minigene consisting of TRRAP to evaluate the process of scaRNA28 synthesis

**Figure 1.**
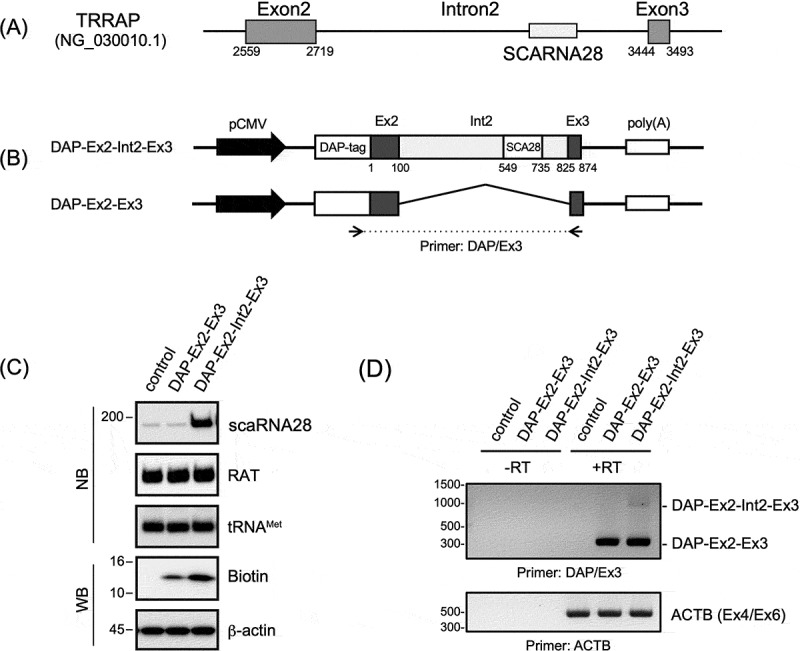
(A) Schematic representation of exon 2 and exon 3 of *TRRAP* containing the *SCARNA28* gene. The number indicating the location of each site is described based on NG_030010.1. (B) Schematic representations of minigenes expressing DAP-tagged exon 2 and exon 3 protein and scaRNA28 (DAP-Ex2-Int2-Ex3) or those without scaRNA28 (DAP-Ex2-Ex3). Primer sets (DAP/Ex3) used for RT-PCR analysis are indicated by arrows, and the amplified fragment is indicated by the dotted line. CMV promoter (pCMV) and poly(A) signal derived from the plasmid backbone are indicated. (C) Northern and western blotting of indicated RNAs and proteins, respectively. RAT RNA was used as a transfection control and tRNA^Met^ as the loading control for northern blotting (NB). DAP-tagged protein (Biotin) was detected using stabilized streptavidin-HRP conjugate. β-actin was used as a loading control for western blotting (WB). Molecular weight markers are indicated at the left of each blot. (D) RT-PCR analysis of the transcripts of the transfected minigenes. Transcript encoding DAP-tagged exon 2 and exon 3, and DAP-tagged exon 2, intron 2, and exon 3 are indicated on the right side of the signals. ACTB was used as a loading control. Primer sets used in the figures are indicated at the bottom of each gel band.

*SCARNA28* gene locus is in intron 2 of *TRRAP* between exons 2 and 3 (NG_030010.1) (Figure 1A). scaRNA28 is likely generated depending on the processing of intron 2, which is spliced from transcripts comprising the region from exon 2 to exon 3 of *TRRAP* as the host gene. To confirm the process of scaRNA28 synthesis from *TRRAP* transcripts, we constructed a minigene comprising the regions of exon 2, intron 2, and exon 3 of *TRRAP* following the sequence-coding double affinity purification (DAP) tag, comprising His6, Biotinylated sequence, and FLAG (Figure 1B, shown as DAP-Ex2-Int2-Ex3, and Supplementary Figure S1) [25]. The DAP-Ex2-Int2-Ex3 minigene was transfected into 293T cells, and the expression of DAP-tagged proteins comprising exons 2 and 3 of *TRRAP* and scaRNA28 were detected by western and northern blotting with specific probes for scaRNA28, respectively (Figure 1C and Supplementary Figure S2). pRcU6PT expressing RAT-tagged RNA was co-transfected with minigenes as transfection controls. RT-PCR analysis revealed a pre-mRNA containing intron 2 (indicated as DAP-Ex2-Int2-Ex3) to be spliced, which was detected in the transcripts of the DAP-Ex2-Int2-Ex3 minigene but not in the control minigene (DAP-Ex2-Ex3) comprising exons 2 and 3 of *TRRAP* (Figure 1D). Thus, we constructed a minigene-expressing scaRNA28 to evaluate the relationship between splicing events, intron processing, and scaRNA28 synthesis.

### scaRNA28 was synthesized even when the intron 2 splicing of TRRAP was suppressed

**Figure 2.**
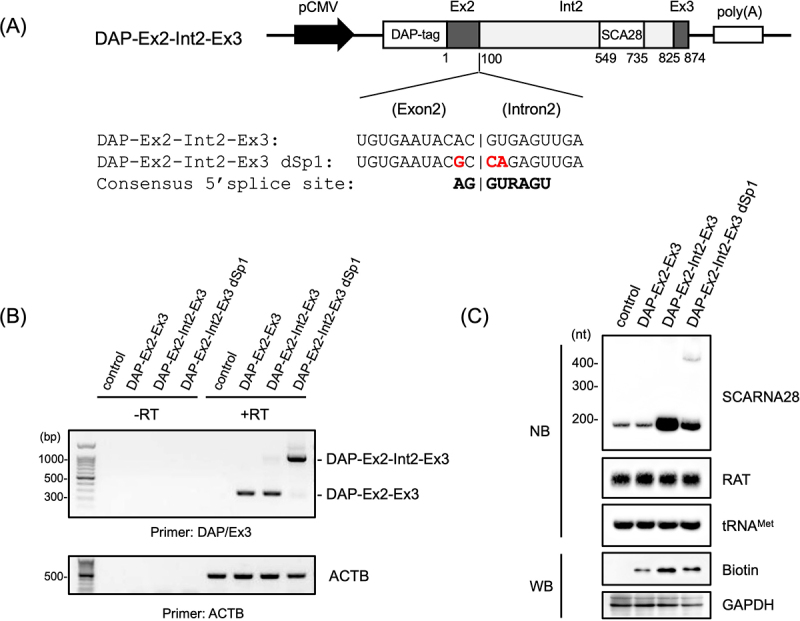
(A) Schematic representation of minigenes encoding transcripts with wild-type and mutation in splicing site (dSp). Consensus sequence of typical 5′-splice site is indicated. (B) RT-PCR analysis of the transcripts of transfected minigenes. Transcript encoding DAP-tagged exon 2 and exon 3, and DAP-tagged exon 2, intron 2, and exon 3 are indicated on the right side of the signals. ACTB was used as a loading control. Primer sets used in the figures are indicated at the bottom of each gel band. (C) Northern blotting (NB) and western blotting (WB) of indicated RNAs and proteins. RAT RNA was used as the transfection control and tRNA^Met^ as the loading control for northern blotting. DAP-tagged protein (biotin) was detected using stabilized streptavidin-HRP conjugate. GAPDH was used as the loading control for western blotting. Molecular weight markers are indicated at the left of each blot.

To evaluate whether intron 2 splicing of *TRRAP* pre-mRNA contributed the synthesis of scaRNA28, we constructed another minigene with mutations at the exon 2/intron 2 junction (Figure 2A). Based on the information of the 5′ splicing consensus sequence, which was reported by the genome-wide splicing site analysis [26], three bases in the vicinity of the splicing junction were mutated to disrupt the splicing site recognition of exon 2/intron 2 junction (Figure 2A). These minigenes were transfected into 293T cells, and transcripts encoding DAP-tagged proteins and scaRNA28 were detected using RT-PCR and northern blotting. RT-PCR analysis showed that most transcripts derived from the mutated minigene (DAP-Ex2-Int2-Ex3 dSp1) consisted of exon 2, intron 2, and exon 3 with a drastically reduced number of transcripts in the spliced intron 2 form, indicating that intron 2 splicing events suppressed in the transcripts from the DAP-Ex2-Int2-Ex3 dSp1 minigene (Figure 2B). A very weak band with 300 bp from DAP-Ex2-Int2-Ex3 dSp1 minigene was a newly spliced isoform with exon 2(1–59) and exon 3, which was generated by disruption of exon 2/intron 2 recognition. In contrast, scaRNA28 was generated from the DAP-Ex2-Int2-Ex3 dSp1 minigene, although the scaRNA28 levels were lower than those derived from cells with the DAP-Ex2-Int2-Ex3 minigene (Figure 2C). Furthermore, a possible precursor of scaRNA28 (400 nt) was detected only in cells transfected with the DAP-Ex2-Int2-Ex3 dSp1 minigene (Figure 2C). The transfection of DAP-Ex2-Int2-Ex3 dSp2 minigene with the mutated 5′ and 3′-splice sites in intron 2 showed an increased scaRNA28 precursor compared to DAP-Ex2-Int2-Ex3 dSp1, although it also slightly generated another spliced isoform consisting of the 1–59 and 796–874 region (Supplementary Figure S3A – C). These results suggest that intron 2 splicing of transcripts from the *TRRAP* minigene is not necessarily required for generating scaRNA28 and that there are other pathways for scaRNA28 synthesis, including endonucleolytic processing of intron-containing transcripts.

scaRNA28 contains a UG repeat sequence within its internal sequence [10], which is the target for TDP-43 binding and responsible for localization into the Cajal body, in which scaRNAs function as guide RNAs for chemical modification containing 2′-*O*-methylation and pseudouridylation of U snRNAs [8,11,27]. To investigate whether the UG repeat sequence of scaRNA28 contributes to the intron 2 splicing of *TRRAP* transcripts and the synthesis of scaRNA28, we constructed a UG repeatless minigene by replacing the UG repeat with the UC repeat sequence of scaRNA28 (Supplementary Figure S3D). Transfection of the UG repeatless minigene (DAP-Ex2-Int2-Ex3 p(CU)_11_) into 293T cells did not show any effect on the efficiency of intron 2 splicing compared with that of DAP-Ex2-Int2-Ex3 minigene (Supplementary Figure S3E), whereas the amount of scaRNA28 decreased by 20 % (Supplementary Figure S3F and S3G). These results indicate that the UG repeat sequence of scaRNA28 is responsible for its stability but not for the splicing of *TRRAP* transcripts.

### Promoterless minigene comprising exon 2, intron 2, and exon 3 of TRRAP generated scaRNA28

**Figure 3.**
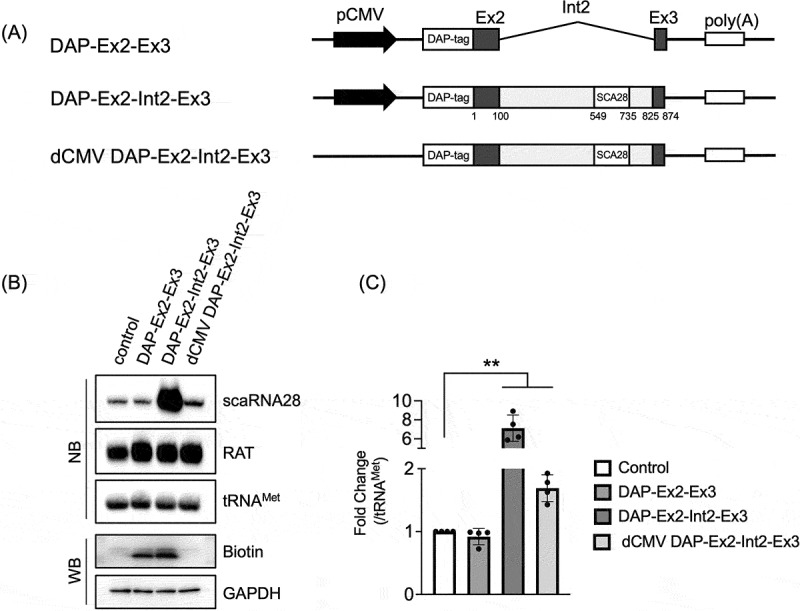
(A) Schematic representation of minigenes encoding DAP-tagged exon 2 and exon 3 protein (DAP-Ex2-Int2-Ex3) with or without (ΔCMV) CMV promoter. (B) Northern blotting (NB) and western blotting (WB) of indicated RNAs and proteins. RAT RNA was used as the transfection control and tRNA^Met^ as the loading control for northern blotting. DAP-tagged protein (Biotin) was detected using stabilized streptavidin-HRP conjugate. GAPDH was used as the loading control for western blotting. (C) The graph shows relative scaRNA28 levels indicated in each lane in (B). The signal of scaRNA28 was normalised to that of tRNA^Met^. Data represent the mean ± standard deviation (SD) of four independent experiments. **p < 0.01 (paired *t*-test).

Considering that intron 2 splicing of transcripts from the *TRRAP* minigene is not indispensable for generating scaRNA28, we further investigated the possibility that transcripts not derived from host gene transcription may also be involved in scaRNA28 synthesis. To investigate the minimum requirement of minigene composition for scaRNA28 expression, we constructed a promoterless minigene in which the transcripts derived from the CMV promoter were not expressed; consequently, the proteins were not synthesized (Figure 3A,B). Nevertheless, the cells transfected with promoterless minigene (dCMV DAP-Ex2-Int2-Ex3) expressed 1.7-fold scaRNA28 of endogenous scaRNA28 in control cells transfected with empty plasmid, indicating that the promoterless minigene expressed small amounts of scaRNA28 (Figure 3B,C, and Supplementary Figure S4A – C). These results indicate that the region from exon 2 to exon 3 of *TRRAP* contains a potential ITU from which the host gene promoter-independent transcripts were generated, leading to the synthesis of scaRNA28 using a different mechanism.

**Figure 4.**
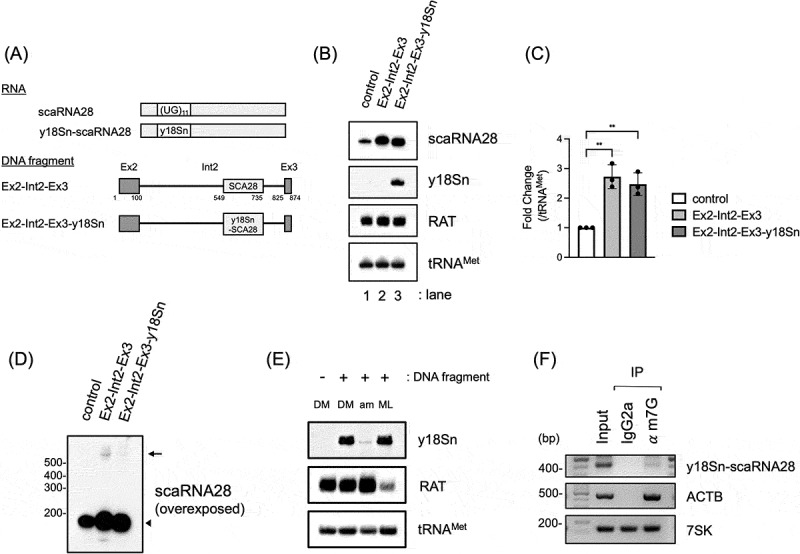
(A) Schematic representation of DNA fragments consisting of exon 2, intron 2, and exon 3 of *TRRAP*. Schematic representation of wild-type and y18Sn-tagged scaRNA28 (top), and DNA fragments, encoding scaRNA28 or y18Sn-scaRNA, consisting of exon 2, intron 2, and exon 3 of *TRRAP* (bottom). (B) Northern blotting of indicated RNAs from the cells transfected DNA fragments. RAT RNA was used as the transfection control and tRNA^Met^ as the loading control for northern blotting. The probes used are indicated on the right side of the blot. (C) The graph shows the scaRNA28 levels indicated in each lane relative to that in lane 1 in (B). The signal of scaRNA28 was normalised to that of tRNA^Met^. Data represent the mean ± SD of three independent experiments. **p < 0.01 (Dunnett’s test). (D) The overexposed image of northern blotting with the probe for scaRNA28 in (B). The putative precursors of scaRNA28 are indicated by an arrow, and mature scaRNA28 and y18Sn-scaRNA28 by an arrowhead. (E) Northern blotting of indicated RNAs from the cells transfected with Ex2-Int2-Ex3-y18Sn, which were treated with α-amanitin (am), or ML-60218 (ML). Dimethyl sulfoxide (DM) was used as a control. RAT RNA was used as a positive control for pol III inhibition by ML-60218. tRNA^Met^ was used as the loading control for northern blotting. The probes used are indicated on the right side of the blot. (F) Immunoprecipitation of m^7^G-capped y18Sn-scaRNA28 precursor using anti-m^7^G antibody. y18Sn-scaRNA28 precursor was detected by PCR with the primer set to amplify the region from Intron 2 of TRRAP to y18Sn.

Further experiments using DNA fragments without extra DNA were conducted to exclude the possibility that other sequences of the promoterless minigene, corresponding to the plasmid backbone or DAP-tagged sequences, were responsible for the production of scaRNA28. We carried out PCR amplification of a DNA fragment consisting of the Ex2-Int2-Ex3 region of *TRRAP* using promoterless minigene (dCMV DAP-Ex2-Int2-Ex3) as a PCR template (Figure 4A, see Methods for detail). We also constructed a minigene-expressing scaRNA28 with a y18Sn RNA tag in its internal sequence to distinguish between minigene-derived scaRNA28 and endogenous scaRNA28 (Supplementary Figure S5A, shown as DAP-Ex2-Int2-Ex3-y18Sn). The y18Sn RNA tag was used to detect various noncoding RNAs, such as U1 and U2 snRNAs [24,28]. The UG repeat sequence of scaRNA28 was replaced with the y18Sn RNA tag sequence, which could be detected using a probe specific to the y18Sn RNA sequence (Supplementary Figure S5B). We transfected DAP-Ex2-Int2-Ex3-y18Sn minigene to 293T cells, and detected y18Sn-tagged scaRNA28 (y18Sn-scaRNA28) without detecting signals from endogenous scaRNA28 (Supplementary Figure S5B). We prepared a DNA fragment coding y18Sn-scaRNA28 by PCR using the CMV promoterless DAP-Ex2-Int2-Ex3-y18Sn minigene as a template DNA (Figure 4A). Ex2-Int2-Ex3 or Ex2-Int2-Ex3-y18Sn DNA fragments were transfected into Flp-In 293 T-Rex (293FTR) cells, and scaRNA28 expression was examined (Figure 4B-D). Cells transfected with Ex2-Int2-Ex3 or Ex2-Int2-Ex3-y18Sn DNA fragments expressed more than 2-fold scaRNA28 compared to that expressed by the cells transfected with control DNA (Figure 4B,C). The expression of y18Sn-scaRNA28 was detected by northern blotting using a probe specific for the y18Sn RNA tag, indicating that the transfected DNA fragments indeed expressed scaRNA28 (Figure 4B). The overexposed northern blot image detected with the scaRNA28 probe suggests that transcripts detected at sizes larger than 500 nt may be precursor transcripts of scaRNA28 derived from DNA fragment-transfected cells (Figure 4D). The levels of transcripts expected to be precursors were probably low because of rapid nucleolytic processing. These results strongly indicate that an ITU for scaRNA28 expression exists in the DNA region between exons 2 and 3 of *TRRAP*.

To further characterize the transcription of scaRNA28 expression from the ITU, we used the transcription inhibitors, α-amanitin as an RNA polymerase II inhibitor and ML-60218 as an RNA polymerase III inhibitor, and analysed the expression of y18Sn-scaRNA28 derived from Ex2-Int2-Ex3-y18Sn DNA fragment by northern blotting. ML-60218 treatment did not affect the expression of y18Sn-scaRNA28. However, the expression of RAT-tagged RNA, which is transcribed by RNA polymerase III under the control of the U6 promoter, was drastically reduced (Figure 4E). In contrast, α-amanitin treatment markedly decreased the y18Sn-scaRNA28 levels (Figure 4E). Since transcripts synthesized by RNA polymerase II are modified with a 7-methylguanosine (m^7^G) cap at the 5′ terminus in a transcriptionally coupled manner, we examined whether the y18Sn-scaRNA28 precursor was modified with an m^7^G cap. Immunoprecipitation assay of m^7^G-capped RNA demonstrated that the y18Sn-scaRNA precursor, which was detected by PCR with a primer set designed upstream of y18Sn-scaRNA28 and y18Sn-scaRNA28, was an m^7^G-capped transcript (Figure 4F). These results indicate that RNA polymerase II transcribes scaRNA28 from a DNA fragment containing ITU.

### Promoter region in the independent transcription unit of scaRNA28 is located within exon 2 and intron 2 of TRRAP

**Figure 5.**
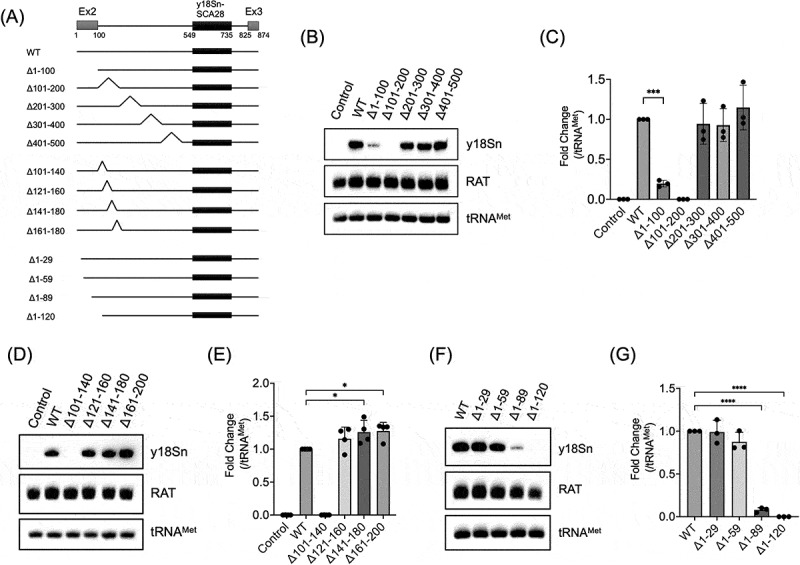
(A) Schematic representation of deletion mutants of DNA fragments encoding y18Sn-scaRNA28. (B, D, F) Northern blotting of indicated RNAs from the cells transfected with DNA fragments. The probes used are indicated on the right side of the blot. RAT RNA was used as the transfection control. tRNA^Met^ was used as the loading control for northern blotting. (C, E, G) The graph shows relative y18Sn-scaRNA28 levels indicated in each lane in (B), (D), or (F). The signal of y18Sn-scaRNA28 was normalised to that of tRNA^Met^. Data represent the mean ± SD of three (C, G) or four (E) independent experiments. *p < 0.05, ***p < 0.001, ****p < 0.0001 (Dunnett’s test).

To identify the promoter region for scaRNA28 expression in the ITU, linker-scanning mutational analysis was performed using mutated Ex2-Int2-Ex3-y18Sn DNA fragments, in which the upstream region of the y18Sn-scaRNA28 coding sequence was sequentially deleted. Five promoterless minigenes with deletion mutants of DAP-Ex2-Int2-Ex3-y18Sn coding y18Sn-scaRNA28 were constructed, and DNA fragments expressing y18Sn-scaRNA28 were prepared by PCR using the minigenes as template DNA (Figure 5A, Δ1–100 to Δ401–500). These DNA fragments were transfected into 293FTR cells and y18Sn-scaRNA28 expression was detected by northern blotting. y18Sn-scaRNA28 were expressed in the cells transfected with DNA fragments of wild-type (WT), Δ1–100, Δ201–300, Δ301–400, and Δ401–500, suggesting that the region corresponding to 101–200 is required for the expression of scaRNA28 (Figure 5B,C). It is also noteworthy that the expression of y18Sn-scaRNA28 was significantly reduced in the Δ1–100 DNA fragments compared to that expressed by the WT DNA fragments (Figure 5B,C). To further limit the promoter region required for scaRNA28 expression in the ITU, eight additional DNA fragments were constructed (Figure 5A, Δ101–140 to Δ1–120). Cells transfected with the DNA fragments of Δ121–160, Δ141–180, or Δ161–200 expressed y18Sn-scaRNA28, but DNA fragments of Δ101–140 did not express y18Sn-scaRNA28 (Figure 5D,E). Furthermore, cells with Δ1–29 or Δ1–59 DNA fragments showed y18Sn-scaRNA28 expression, whereas cells with Δ1–89 showed slight y18Sn-scaRNA28 expression, and Δ1–120 did not show y18Sn-scaRNA28 expression (Figure 5F,G). Collectively, these results indicate that the 101–120 region, which corresponds to the first 20 bases of the intron 2 region of *TRRAP*, is an essential element for scaRNA28 expression, and the 60–120 region has a role as a promoter element for efficient scaRNA28 expression in the ITU. To examine whether the 60–120 region is sufficient for scaRNA28 transcription in the ITU, y18Sn-scaRNA28 expression was confirmed using DNA fragment of Δ1–59,121–500 (Supplementary Figure S6A). Δ1–59,121–500 showed indeed y18Sn-scaRNA28 expression, as WT DNA fragment did, indicating that the 60–120 region has promoter activity for scaRNA28 expression in the ITU (Supplementary Figure S6B and S6C). The luciferase assay using a NanoLuc luciferase (Nluc) – expressing plasmid, which includes the 60–120 region as a promoter (p(60–120)-Nluc), showed that p(60–120)-Nluc increased luciferase intensities compared to the promoter-less (pl)-Nluc, suggesting that the 60–120 region functions as a promoter (Supplementary Figure S6D). To evaluate the effect of the 3′ region on scaRNA28 expression, another DNA fragment with a mutation in the intron 2 and exon 3 junction was constructed (Supplementary Figure S6A; indicated as dSp3). dSp3 showed drastically reduced y18Sn-scaRNA28 expression, suggesting that most of the y18Sn-scaRNA28 expression in the ITU is generated by splicing at the 3′-splice site of intron 2 (Supplementary Figure S6E and S6F).

### E-box DNA sequence is essential for scaRNA28 expression in the independent transcription unit

**Figure 6.**
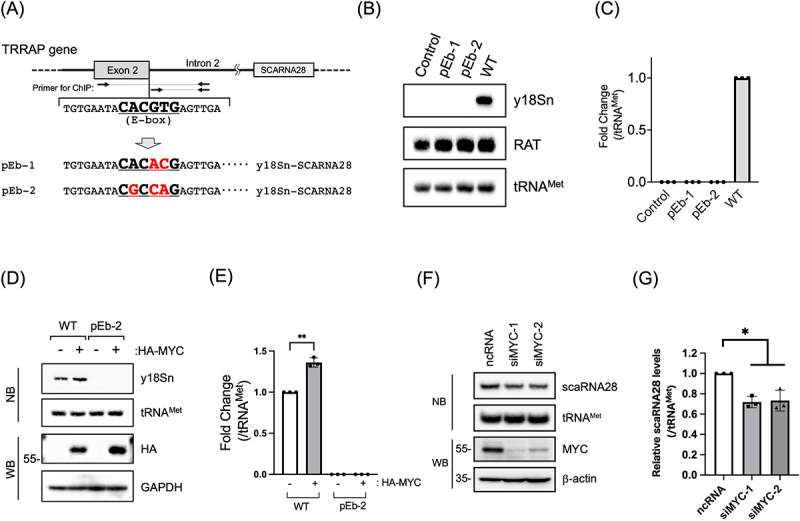
(A) Schematic representation of the putative E-box motif at the exon 2/intron 2 junction of *TRRAP* (top). DNA fragments containing the mutations within E-box motif (pEb-1, pEb-2). (B) Northern blotting of indicated RNAs from the cells transfected with each DNA fragments. The probes used are indicated on the right side of the blot. RAT RNA was used as the transfection control. tRNA^Met^ was used as the loading control for northern blotting. (C) The graph shows the y18Sn-scaRNA28 levels relative to that of the control in (B). The signal of y18Sn-scaRNA28 was normalised to that of tRNA^Met^. (D) HA-MYC and DNA fragments coding Ex2-Int2-Ex3-y18Sn (WT) or Ex2-Int2-Ex3-y18Sn pEb-2 were co-transfected into 293FTR cells. The expression of the indicated RNAs and proteins were detected by northern blotting (NB) and western blotting (WB), respectively. Empty plasmid (pcDNA5/FRT/TO) was used as a transfection control. The probes used are indicated on the right side of the blot. tRNA^Met^ was used as the loading control for northern blotting. HA-MYC was detected with an anti-HA antibody. (E) The graph shows relative scaRNA28 levels in (D). The signals of scaRNA28 were normalized to that of tRNA^Met^. Data represent the mean ± SD of three independent experiments. **p < 0.01 (paired *t*-test). (F) Northern blotting (NB) and western blotting (WB) of the indicated RNAs and proteins, respectively, upon MYC knockdown. Probes and antibodies used are indicated on the right side of the blots. Negative control RNA (ncRNA) was used as the control RNA against MYC siRNA (siMYC-1, siMYC-2). (G) The graph shows relative scaRNA28 levels in (F). The signals of scaRNA28 were normalized to that of tRNA^Met^. Data represent the mean ± SD of three independent experiments. *p < 0.05 (paired *t*-test).

Given that the first 20 bases of the intron 2 region of *TRRAP* are required for scaRNA28 expression and deletion of the 1–89 region within exon 2 drastically reduced scaRNA28 expression from DNA fragments (Figure 5D-G), we investigated the involvement of the DNA sequence in the vicinity of the exon 2/intron 2 junction in scaRNA28 expression in the ITU. To identify possible regulatory elements in the vicinity of the exon 2/intron 2 junction, we used open-access JASPAR software to predict candidate regulatory DNA elements and transcription factors that bind to DNA elements. The JASPAR software predicted the binding of the three transcription factors to the 1–121 regions of the Ex2-Int2-Ex3 DNA sequence with a high score (Supplementary Table S5). Of these, we focused on the DNA sequence of the exon 2/intron 2 junction, which contains a putative E-box motif (CACGTG): MYC:MAX binding sequence (Figure 6A). We prepared two DNA fragments consisting of Ex2-Int2-Ex3 with a mutation in the E-box motif (pEb-1 and pEb-2) and evaluated scaRNA28 expression using these DNA fragments (Figure 6B). The results showed that the cells transfected with the pEb-1 or pEb-2 DNA fragments did not express y18Sn-scaRNA28, indicating that these DNA sequences, especially the fourth and fifth 2 bases within the E-box motif, are essential for scaRNA28 expression in the ITU (Figure 6B,C).

We evaluated the involvement of MYC in scaRNA28 expression in the ITU. Co-transfection of the y18Sn-scaRNA28 DNA fragment and exogenous MYC (HA-MYC) into 293FTR cells showed that HA-MYC expression substantially increased y18Sn-scaRNA28 expression compared to that in cells without HA-MYC expression (Figure 6D,E). Chromatin immunoprecipitation (ChIP) analysis revealed that endogenous MYC bound to the region between exon 2 and intron 2 of endogenous *TRRAP* (Supplementary Figure S7A). These results suggest that MYC is a possible transcriptional regulator of scaRNA28 expression in the ITU. To investigate whether the MYC protein affects total endogenous scaRNA28 levels, we conducted additional experiments by knocking down endogenous MYC expression using siRNA or overexpressing MYC using doxycycline-inducible MYC expression cells. MYC knockdown reduced total endogenous scaRNA28 levels by 30 %, whereas the overexpression of MYC did not show significant changes in endogenous scaRNA28 levels (Figure 6F,G, and Supplementary Figure S7B and S7C). These results suggest that MYC expression affects the total expression of scaRNA28, at least through its role as a transcriptional regulatory factor of scaRNA28 in the ITU.

## Discussion

In this study, we focused on scaRNA28 as a predictive intronic-embedded scaRNA and investigated its synthesis in detail. scaRNA28 was generated even when the splicing of the host gene was suppressed (Figure 2). These results suggest a possible mechanism by which scaRNA28 is excised from unspliced transcripts, in addition to processing spliced intron 2, although in reduced amounts or efficiency compared to scaRNA28 derived from spliced transcripts. scaRNA28 expression was substantially increased compared to that of endogenous scaRNA28, even when the host gene promoterless minigene or DNA fragments consisting of exon 2, intron 2, and exon 3 of *TRRAP* were transfected into cells (Figures 3 and 4), strongly suggesting that scaRNA28 is generated from an ITU in which scaRNA28 is expressed in a manner different from that of the host gene (*TRRAP*). To the best of our knowledge, scaRNA28 is the first scaRNA identified to have dual transcriptional pathways.

scaRNA2, scaRNA17, and scaRNA19 (TERC) have ITUs and possess 7-methylguanosine (m^7^G) cap or 2,2,7-trimethylguanosine cap [17,18]. Although we did not detect scaRNA28 with one of these caps using RNA mass spectrometry analysis of mature scaRNA28 in our previous work [11], in this study we demonstrated that scaRNA28 precursor possesses m^7^G cap by the immunoprecipitation assay of m^7^G-capped RNA (Figure 4F). The putative scaRNA28 precursor with more than 500 nt, expressed from the Ex2-Int2-Ex3 DNA fragment, was detected by northern blotting with the overexposed image (Figure 4D). Thus, these results suggest that in the ITU of scaRNA28, scaRNA28 is transcribed as a precursor and processed into mature scaRNA28. This process may also partially involve a mechanism where scaRNA28 is processed from unspliced transcripts expressed in the DAP-Ex2-Int2-Ex3 dSp1 or dSp2 minigene (Figure 2, and Supplementary Figure S3).

Linker-scanning mutational analysis using DNA fragments consisting of exon 2, intron 2, and exon 3 of *TRRAP* expressing y18Sn-scaRNA28 showed that in the ITU, the 60 bases spanning exon 2 and intron 2 regions had promoter activity for scaRNA28 expression, and the first 20 bases of the intron 2 region were required for scaRNA28 expression; in particular, the first two bases, a part of the E-box motif, were essential for scaRNA28 expression (Figures 5 and 6, and Supplementary Figure S6A-C). In addition, the dSp3 DNA fragment with the 3′-splice site mutated drastically reduced scaRNA28 expression, raising the possibility that scaRNA28 expression in the ITU was generated by co-transcriptional splicing (Supplementary Figure S6D and S6E). The Δ1–100 DNA fragment expressed scaRNA28 (Figure 5B), suggesting that 5′-splicing at the canonical 5′-splice site of intron 2 is not necessary for the production of scaRNA28 in the ITU. Since the Δ1–59,121–500 DNA fragment expressed scaRNA28 (Supplementary Figure S6B), the 5′-splice site might exist in the region of 501–548 or immediate downstream of the 5′ transcription start site in the first 20 bases of the intron 2 region.

Predictive analysis of transcription factors raised the possibility that MYC binds to the E-box motif in the ITU of scaRNA28. MYC overexpression increased scaRNA28 expression in the Ex2-Int2-Ex3-y18Sn DNA fragment (Figure 6D,E), and the potential for MYC to bind to the E-box motif of the ITU of scaRNA28 was demonstrated by the ChIP assay (Supplementary Figure S7A). These results strongly suggest that MYC positively regulates scaRNA28 expression in the ITU of scaRNA28. As for the involvement of MYC in the total scaRNA28 levels, MYC knockdown resulted in a 30 % decrease in total scaRNA28 levels (Figure 6F,G). This does not exclude the possibility that MYC is involved in the transcription of host genes but indicates that for up to 30 % of scaRNA28, the expression level of ITU-derived scaRNA28 may be affected. MYC overexpression using doxycycline-inducible MYC expression cells (Supplementary Figure S7B and S7C) or transient overexpression of MYC in 293FTR cells (data not shown) did not affect total scaRNA28 levels. This may suggest that the MYC-inducing effect of scaRNA28 on the endogenous ITUs of scaRNA28 is already saturated in 293FTR cells or suppressed by competition with host gene transcription. In this study, we focused only on MYC, an E-box motif-binding protein, based on the results of JASPAR prediction for the regulation of scaRNA28 expression in the ITU of HEK293-derived cell lines. On the other hand, whether scaRNA28 expression in the ITU is regulated by similar processes and transcription factors in various cell types (e.g. terminally differentiated cells, stem cells, and cancer cells) is a subject for future analysis.

scaRNA28 acts as a guide RNA to modify 2′-*O*-methylation of U47 in U2 snRNA in the Cajal body [10]. We demonstrated that U47 methylation of U2 snRNA is almost 100 % modified in our previous study [11], implying the need for a system that provides scaRNA28 that meets sufficient U2 snRNA modifications, regardless of the host gene expression levels. The expression levels of most intronic-embedded snoRNAs are not correlated with those of their host genes [20,29–31]. No correlation was observed between TRRAP and scaRNA28 expression (Supplementary Figure 8) [32]. This suggests that the biological significance of the ITU of scaRNA28 may lie in the need for stable expression of scaRNA28. In addition to the canonical functions of scaRNAs as guide RNA in RNA modification, some scaRNAs and snoRNAs play distinct roles from canonical functions. scaRNA2, which functions in U2 modification, plays a role in DNA repair as a DNA damage response [33], and SNORA13, a snoRNA, plays a role in the p53 pathway through a non-canonical mechanism distinct from its role in guiding ribosomal RNA (rRNA) modification [34]. Although there are no current reports on a non-canonical mechanism of scaRNA28 that is distinct from its role in guiding U2 snRNA modification, it is possible that scaRNA28 expression from ITU may be used for other cellular processes in response to stress or local needs. The pursuit of physiological functions of scaRNA28, which are different from canonical functions, will be the focus of future investigations.

Similar to scaRNAs, most snoRNAs act as guide RNAs for chemical modification (pseudouridylation or 2′-*O*-methylation) of rRNA during rRNA biogenesis [35]. snoRNA expression levels vary, especially in tumours; however, the mechanisms underlying their regulation remain unclear [21,22,36]. Using genome-wide analysis of the MYC-binding site, Herter et al. showed that MYC acts as a master regulator of snoRNP biogenesis by directly binding to a subset of snoRNA genes in both Drosophila and vertebrates [37]. Although it is still unclear whether the snoRNAs identified by Herter et al. have ITUs or whether there is a correlation between these snoRNAs and host gene expression levels, these reports imply that MYC possibly differentially regulates snoRNA expression, which is unrelated to the regulation of host gene expression. Our findings provide insights into the mechanism by which non-coding RNA containing scaRNAs and snoRNAs are regulated in a manner unrelated to host gene expression under a variety of biological conditions, such as tumour formation.

## Supplementary Material

Supplemental Material

## Data Availability

Raw data for scaRNA28 and host abundance in human tissues and cell lines were downloaded from snoDB 2.0 (https://bioinfo-scottgroup.med.usherbrooke.ca/snoDB/) [32], reported in the supplementary materials of this paper. The source datasets of the raw data are GSE126797 for Ovary, Breast and Prostate, GSE157846 for Testis, Skeletal Muscle, Liver and Brain, GSE99065 for SKOV, GSE209924 for HCT116, MCF7, PC3, TOV112D, SRX1426160 for Universal Human RNA (UHR), and SRX1426193 for Human Brain Reference (HBR). Sanger sequencing raw file analysed in Figure 2B was provided in Supplementary Data 1.
