# Supplementary figures and images for: Dual synthesis pathways of scaRNA28 via intronic processing of transformation/transcription domain-associated protein transcripts and a novel independent transcription unit
Source: RNA Biol. 2025 May 30;22(1):1–12. doi: 10.1080/15476286.2025.2513133 (PMC12150615; doi:10.1080/15476286.2025.2513133)

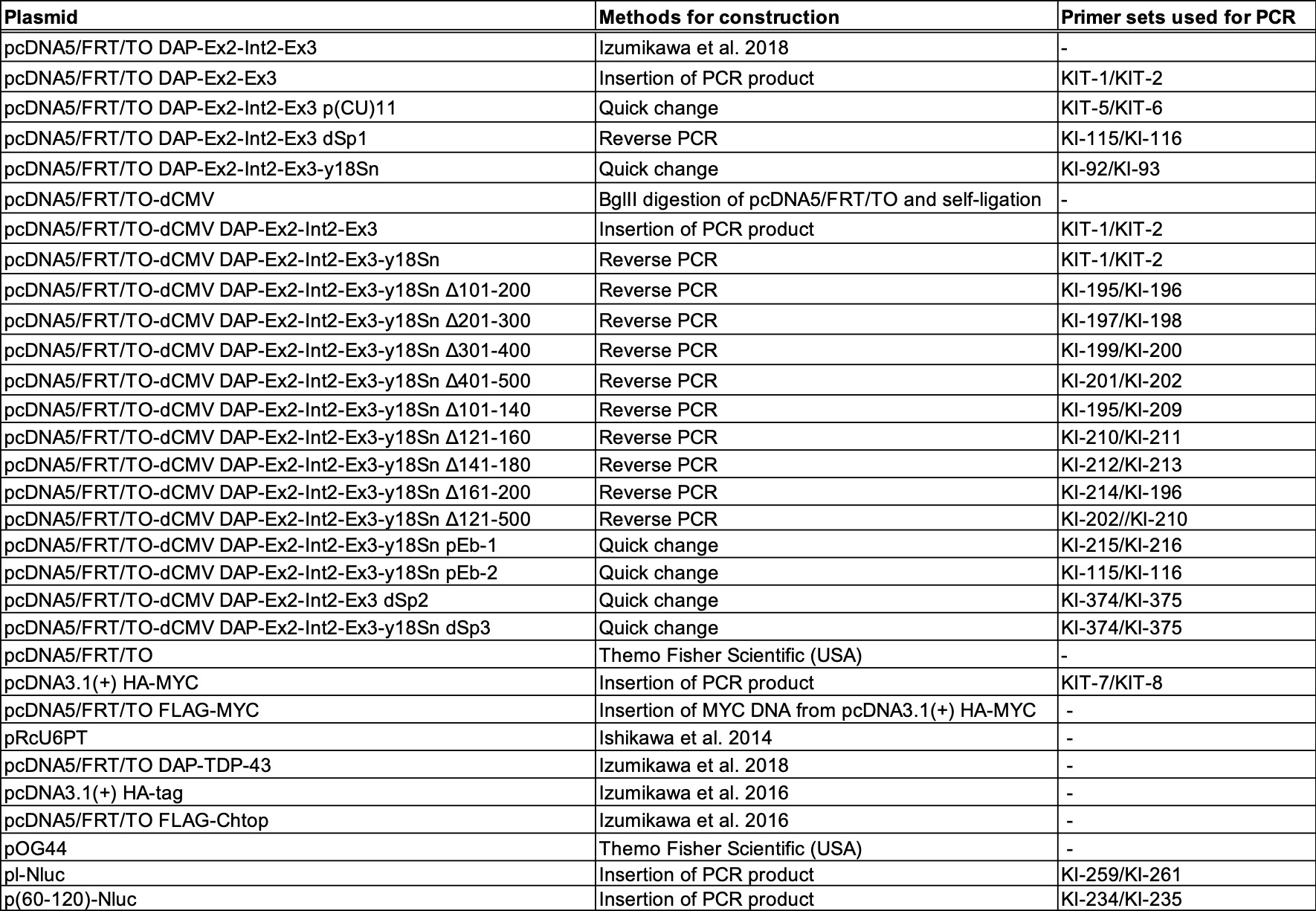

Supplement: Supplemental Material [file KRNB_A_2513133_SM4835.zip › Sup_Table_S3.jpg]

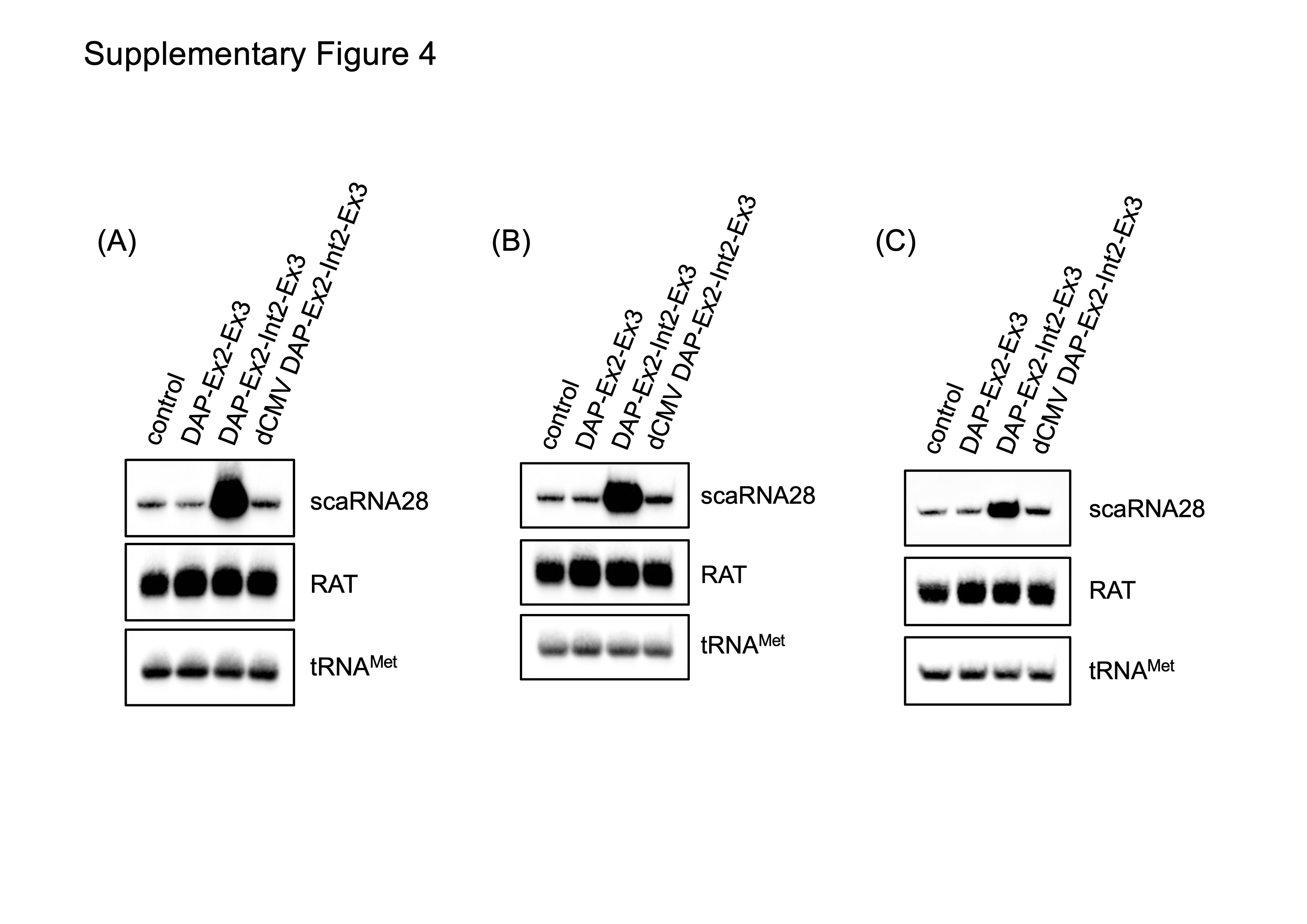

Supplement: Supplemental Material [file KRNB_A_2513133_SM4835.zip › Supple_FigS4.jpg]

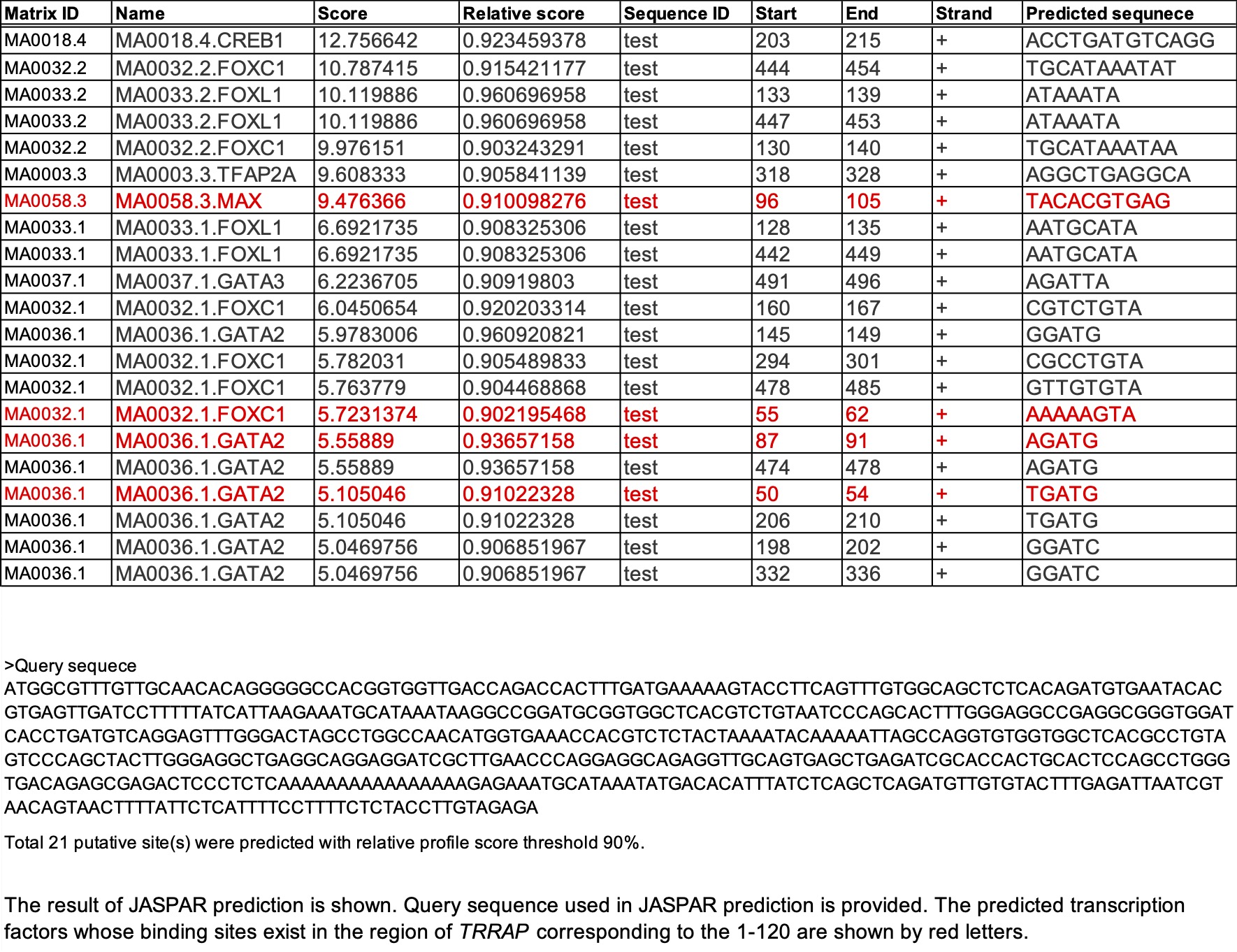

Supplement: Supplemental Material [file KRNB_A_2513133_SM4835.zip › Sup_Table_S5.jpg]

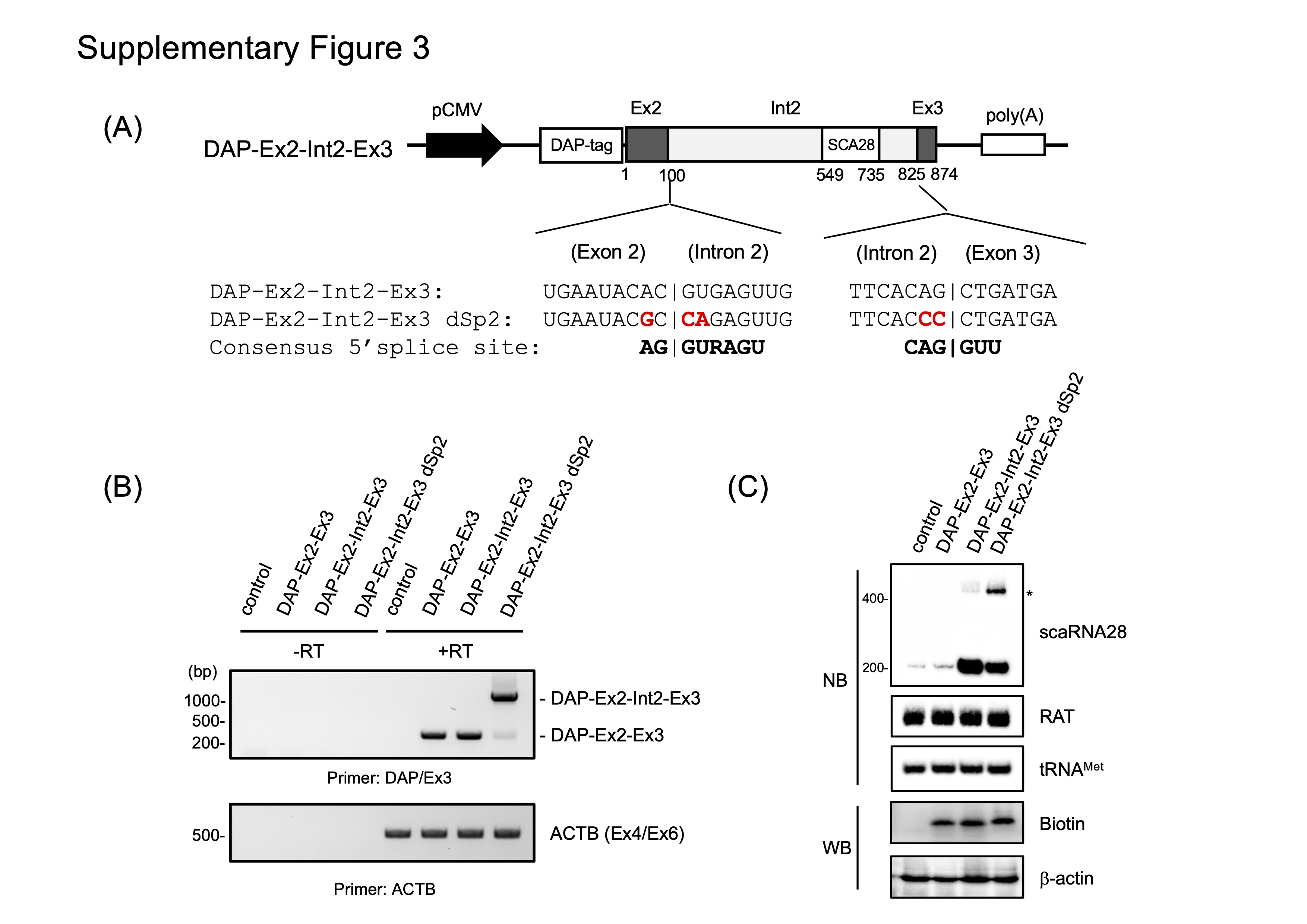

Supplement: Supplemental Material [file KRNB_A_2513133_SM4835.zip › Supple_FigS3.jpg]

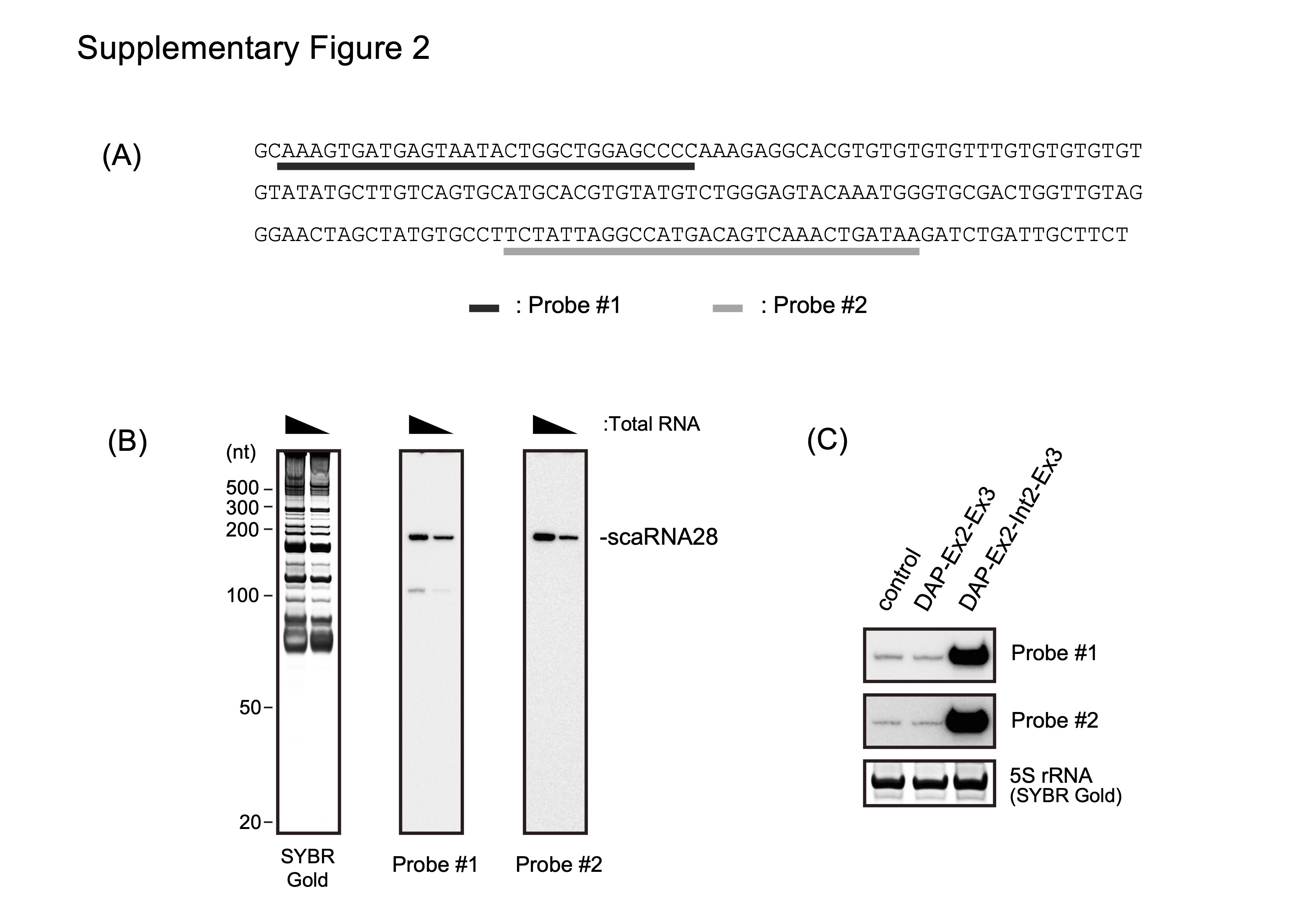

Supplement: Supplemental Material [file KRNB_A_2513133_SM4835.zip › Supple_FigS2.jpg]

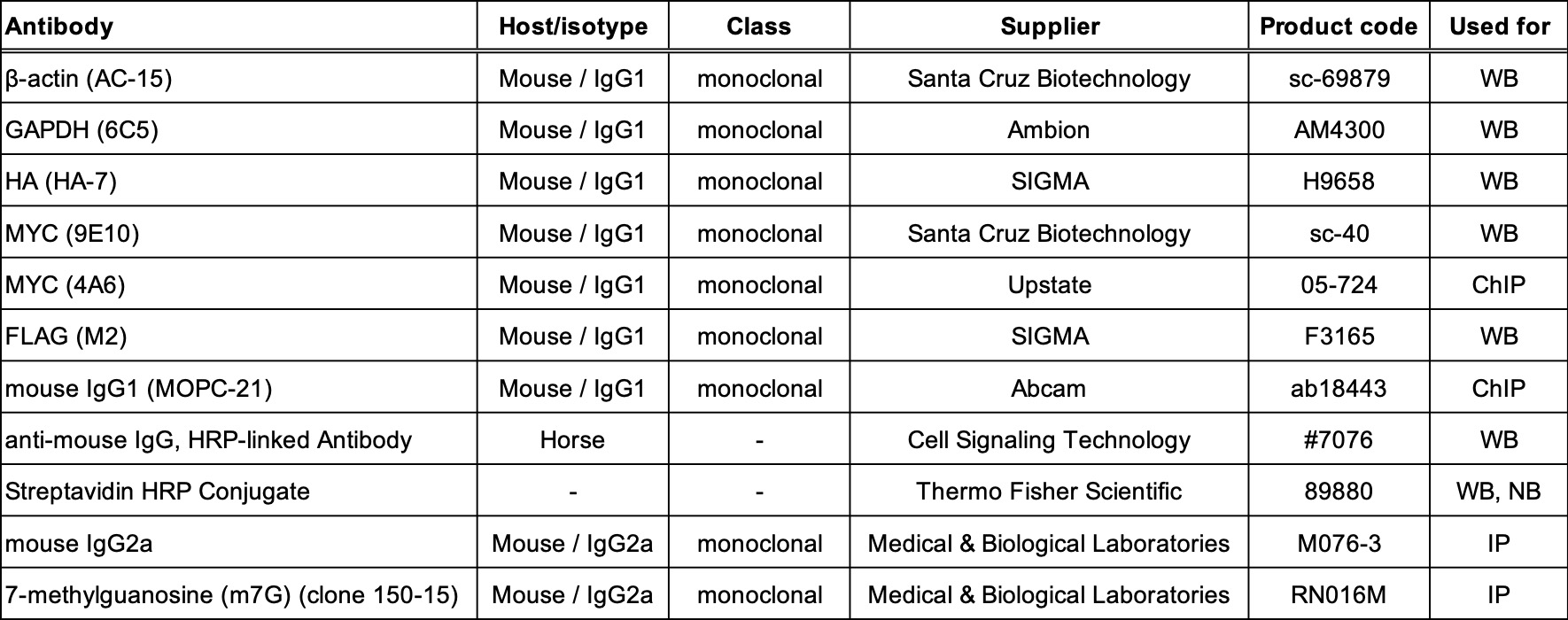

Supplement: Supplemental Material [file KRNB_A_2513133_SM4835.zip › Sup_Table_S1.jpg]

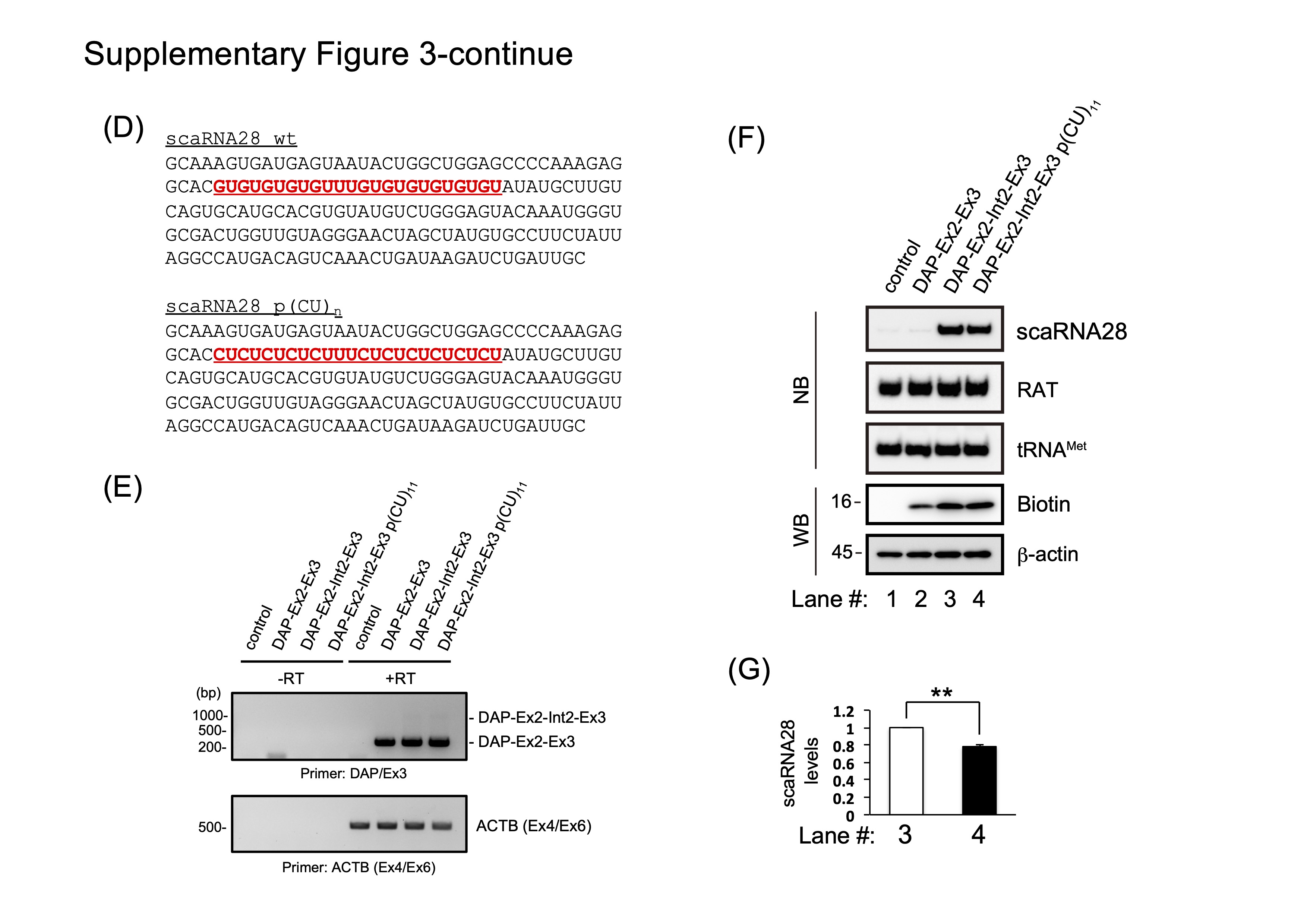

Supplement: Supplemental Material [file KRNB_A_2513133_SM4835.zip › Supple_FigS3-2.jpg]

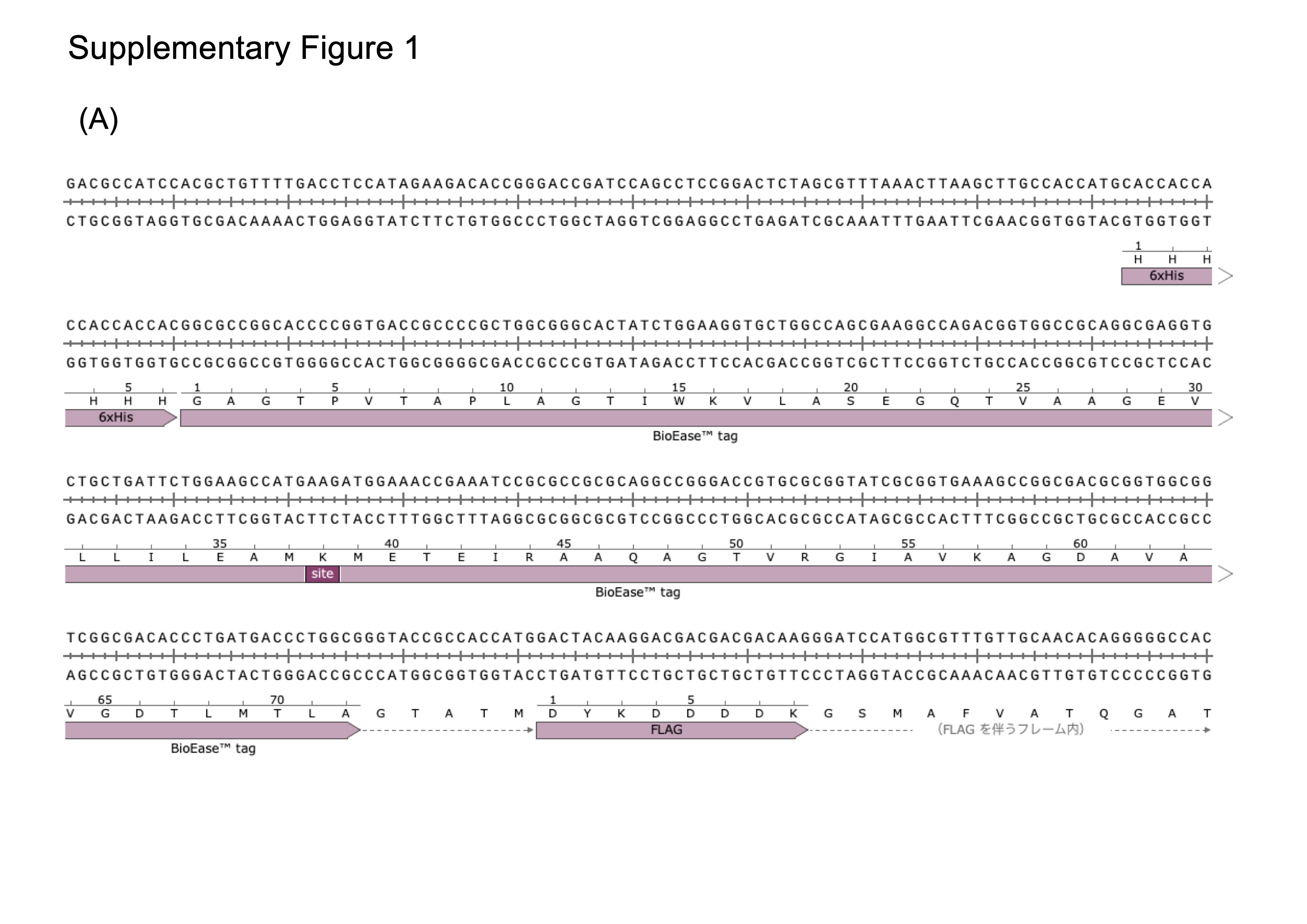

Supplement: Supplemental Material [file KRNB_A_2513133_SM4835.zip › Supple_FigS1.jpg]

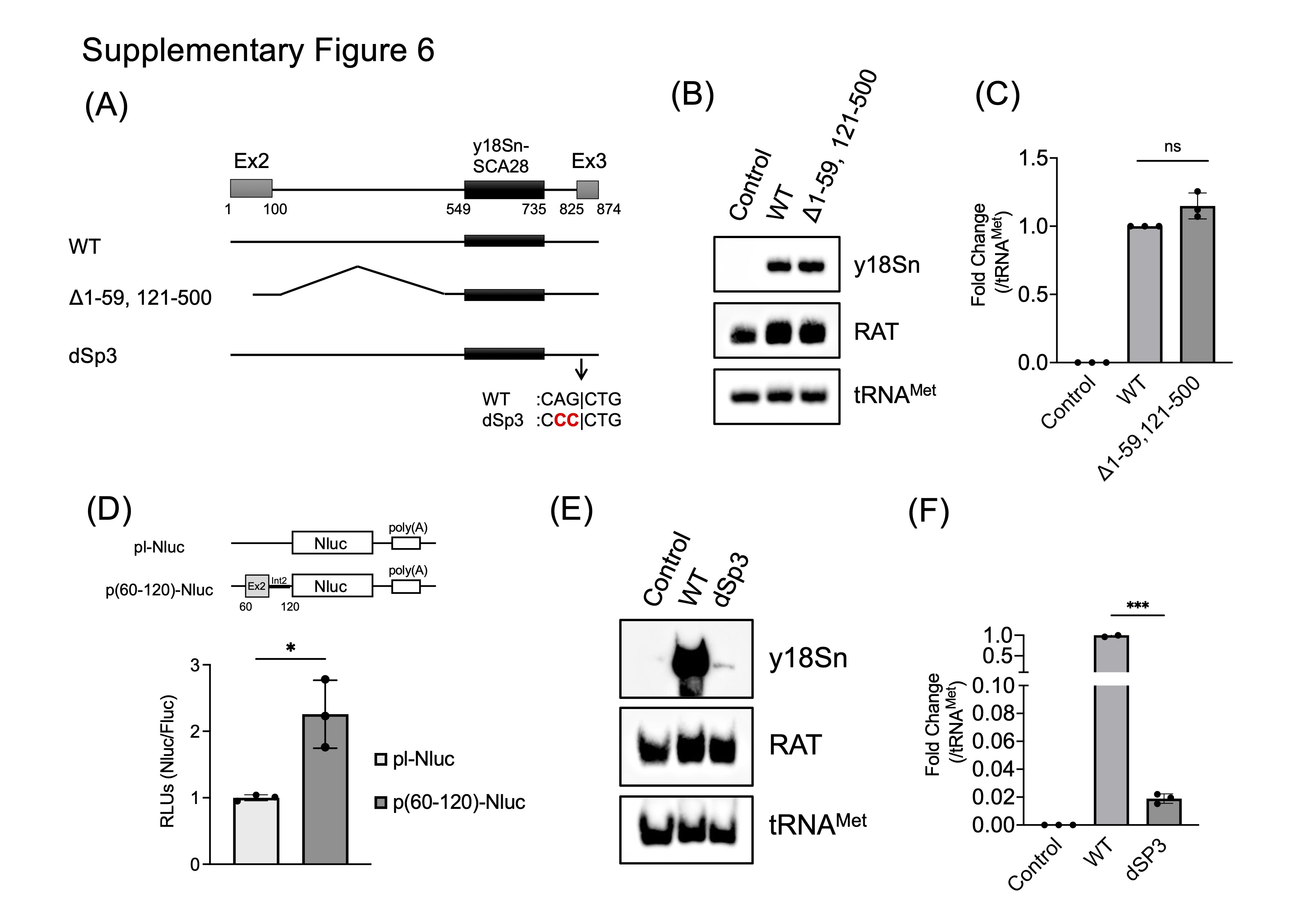

Supplement: Supplemental Material [file KRNB_A_2513133_SM4835.zip › Supple_FigS6.jpg]

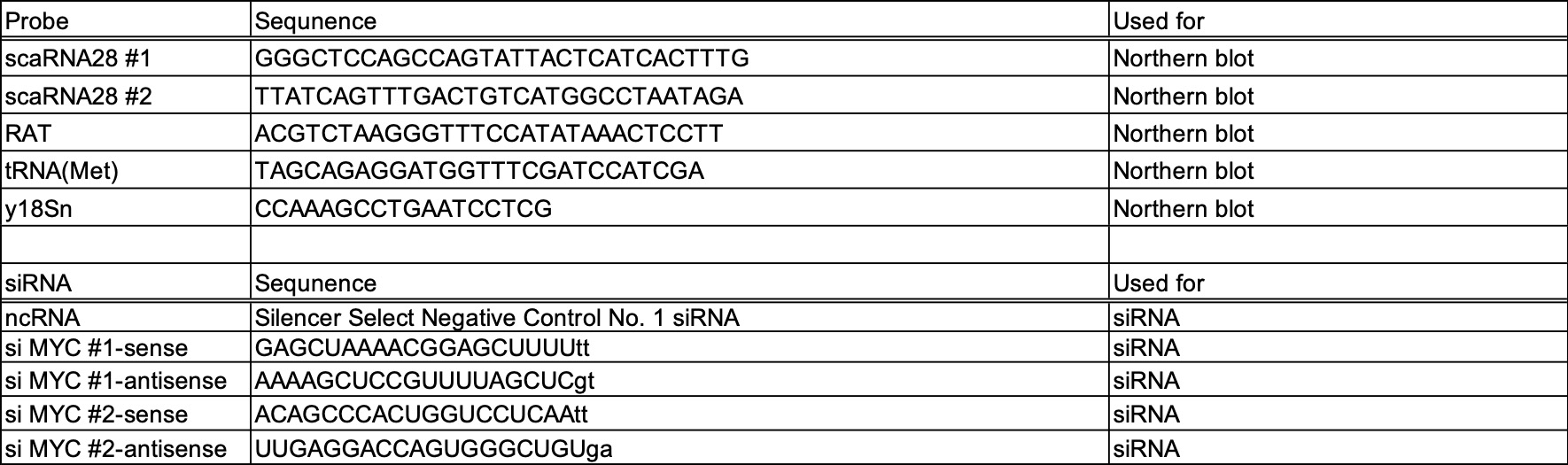

Supplement: Supplemental Material [file KRNB_A_2513133_SM4835.zip › Sup_Table_S2-2.jpg]

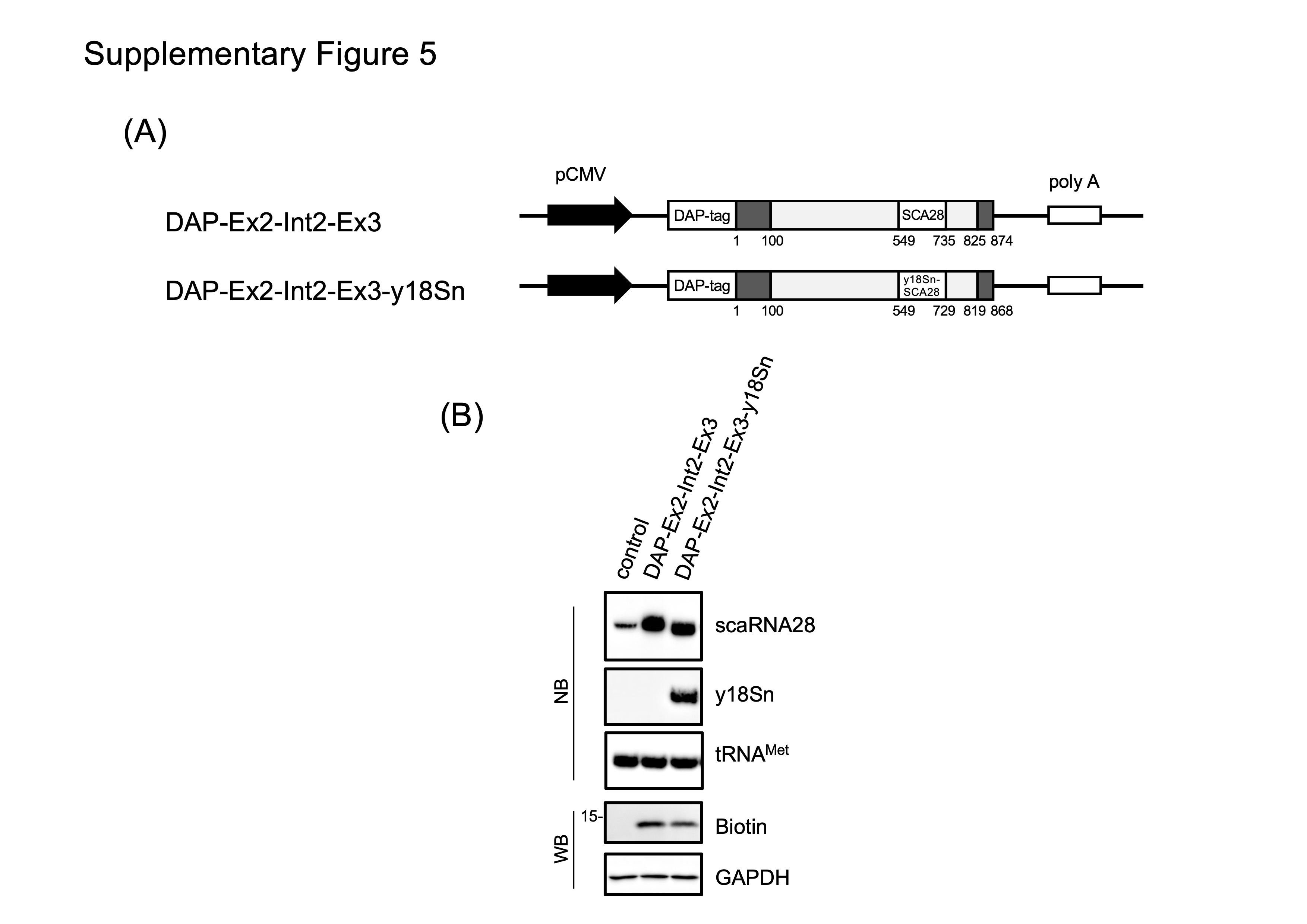

Supplement: Supplemental Material [file KRNB_A_2513133_SM4835.zip › Supple_FigS5.jpg]

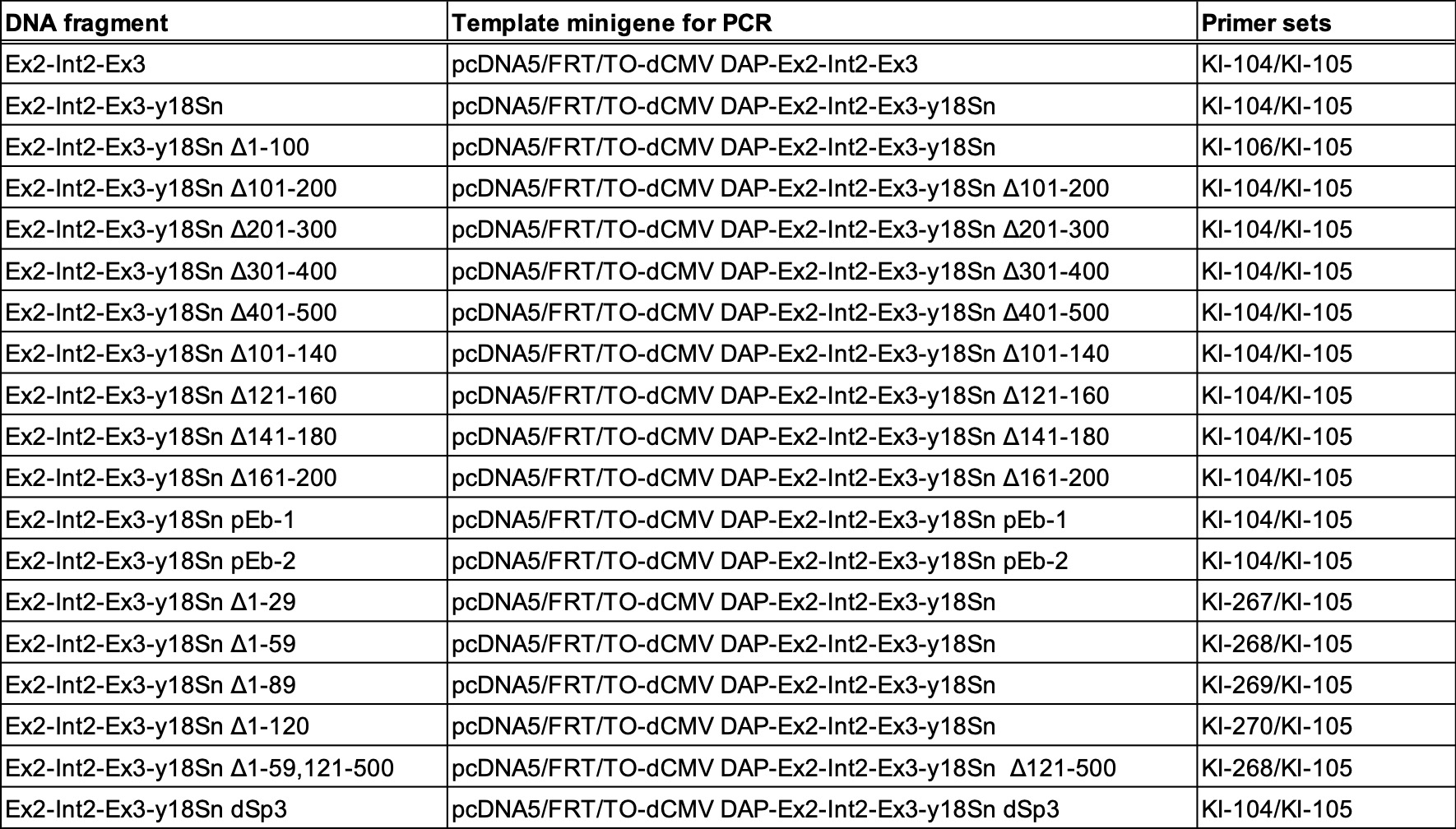

Supplement: Supplemental Material [file KRNB_A_2513133_SM4835.zip › Sup_Table_S4.jpg]

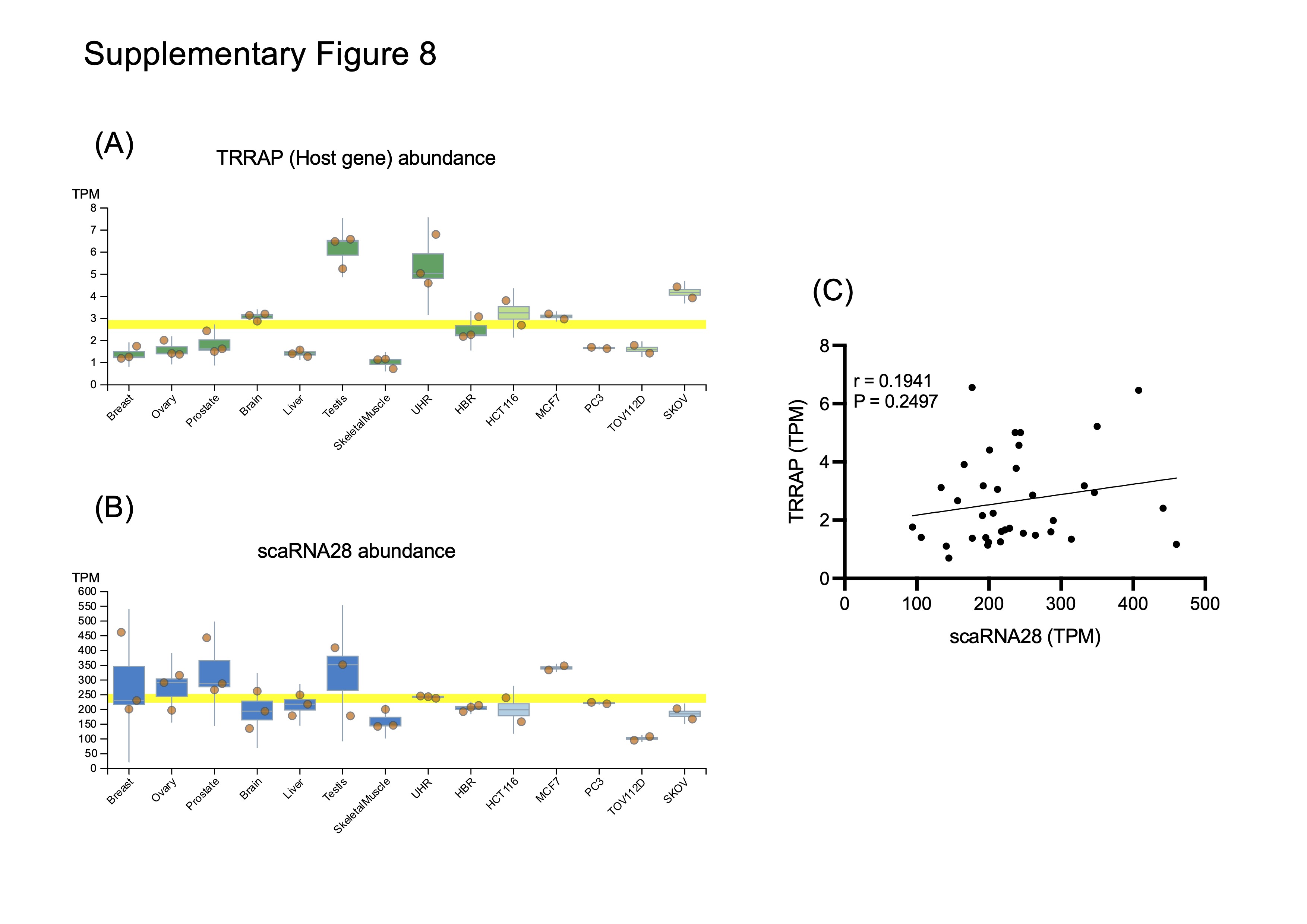

Supplement: Supplemental Material [file KRNB_A_2513133_SM4835.zip › Supple_FigS8.jpg]

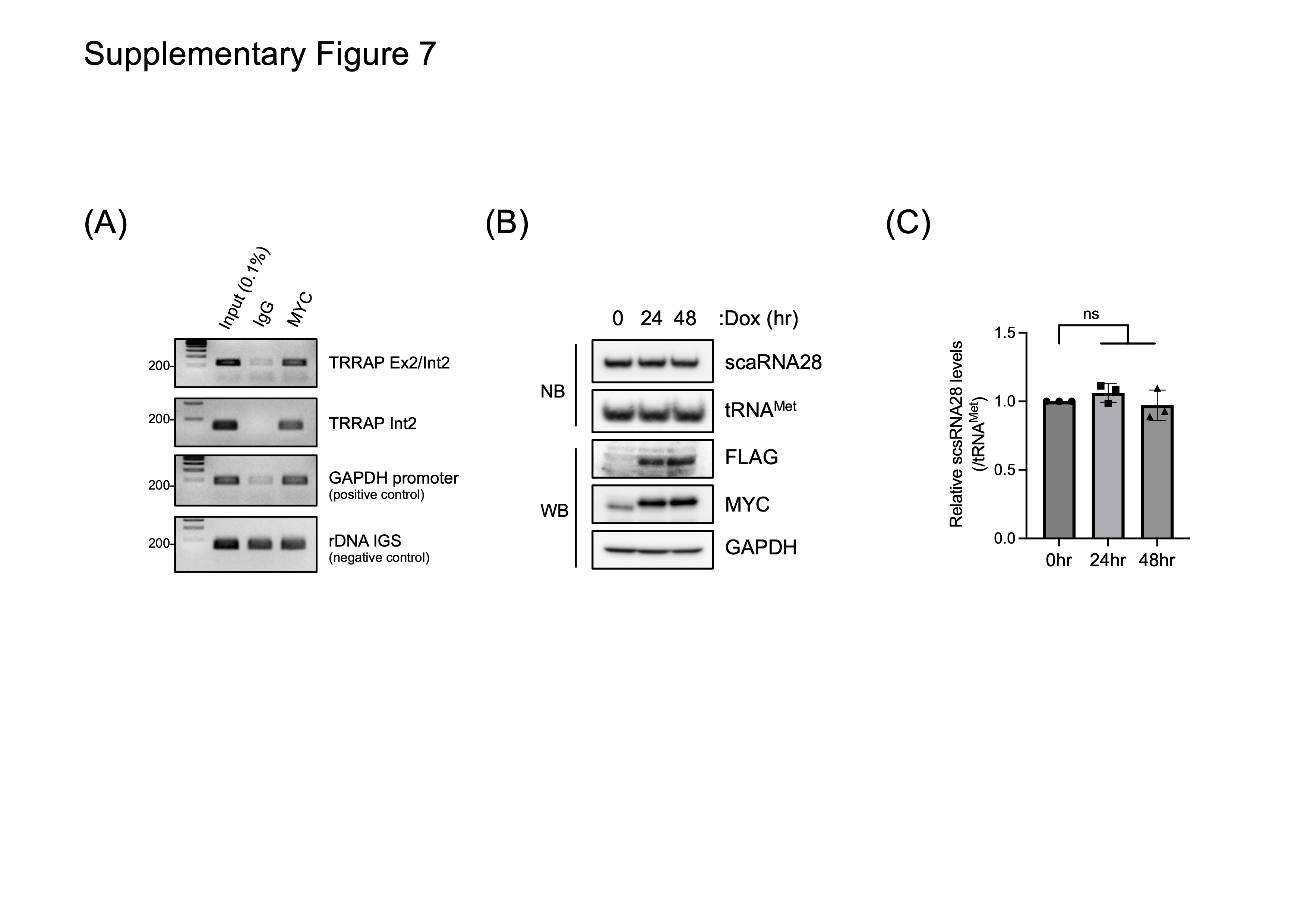

Supplement: Supplemental Material [file KRNB_A_2513133_SM4835.zip › Supple_FigS7.jpg]

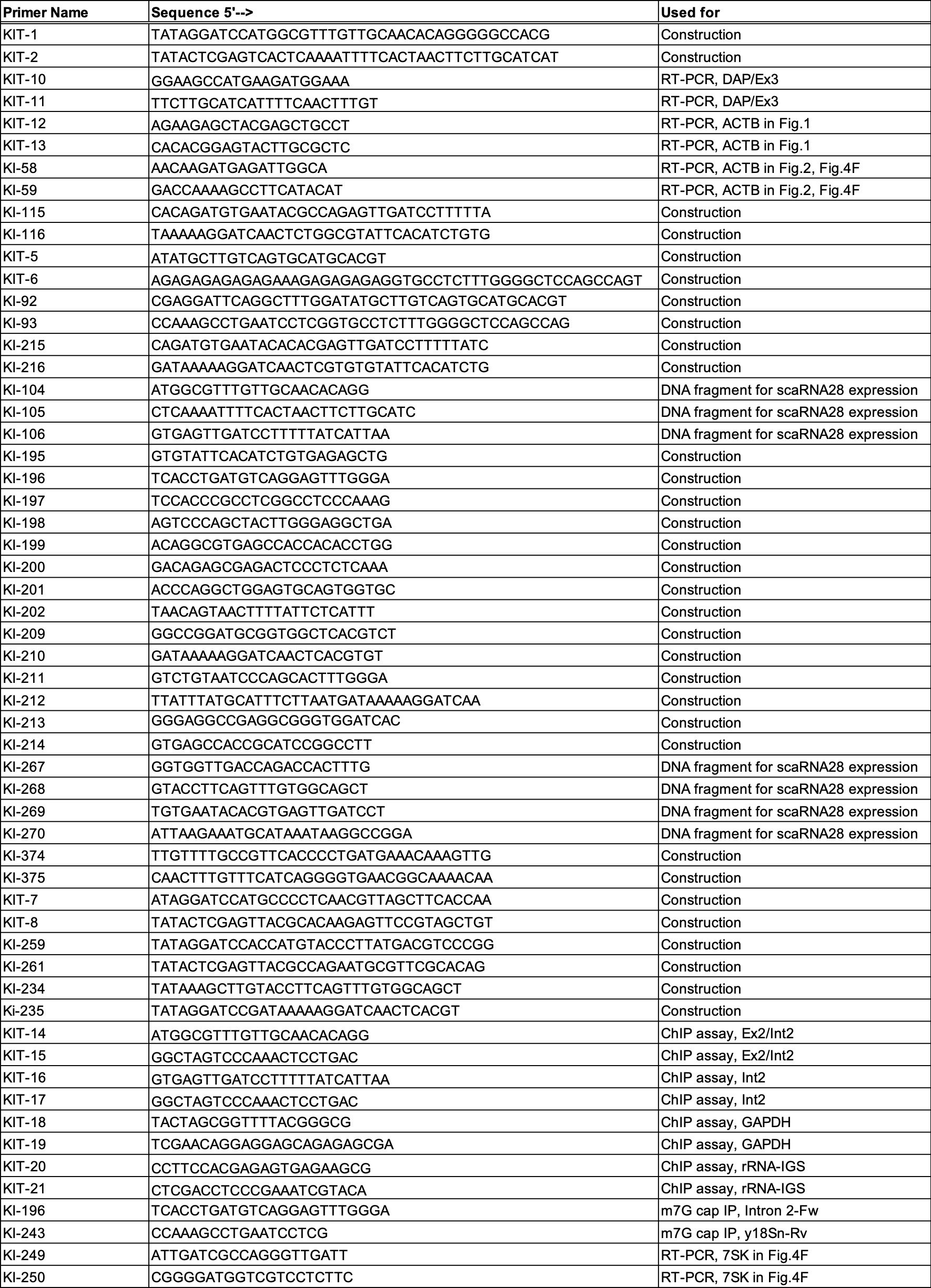

Supplement: Supplemental Material [file KRNB_A_2513133_SM4835.zip › Sup_Table_S2.jpg]
